# *Enterococcus faecalis* Extracellular Vesicles Deliver the Bacterial GTPase Obg to Hijack mTOR Signalling in Hepatocellular Carcinoma

**DOI:** 10.1002/jev2.70323

**Published:** 2026-06-17

**Authors:** Ning Ma, Xiaoshan Xie, Jiarui Wang, Zhikai Zheng, Huilin Jin, Xijie Chen, Xiaoling Huang, Haidan Luo, Yue Wei, Qihao Pan, Boyu Zhang, Jiaying Zheng, Peng Zhang, Fenghai Yu, Xue Liu, Zhi‐Min Zhang, Zhongguo Zhou, Xiangqi Meng, Mong‐Hong Lee

**Affiliations:** ^1^ Guangdong Provincial Key Laboratory of Colorectal and Pelvic Floor Diseases, The Sixth Affiliated Hospital Sun Yat‐sen University Guangzhou China; ^2^ Key Laboratory of Human Microbiome and Chronic Diseases, Ministry of Education Sun Yat‐sen University Guangzhou China; ^3^ Biomedical Innovation Center, The Sixth Affiliated Hospital Sun Yat‐sen University Guangzhou China; ^4^ Department of Liver Surgery Sun Yat‐sen University Cancer Center Guangzhou China; ^5^ State Key Laboratory of Oncology in South China, Guangdong Provincial Clinical Research Center for Cancer Sun Yat‐sen University Cancer Center Guangzhou China; ^6^ Department of Hepatobiliary, Pancreatic and Splenic Surgery, The Sixth Affiliated Hospital Sun Yat‐sen University Guangzhou China; ^7^ Department of General Surgery, The Sixth Affiliated Hospital Sun Yat‐Sen University Guangzhou China; ^8^ Department of Obstetrics and Gynecology, The Sixth Affiliated Hospital Sun Yat‐sen University Guangzhou China; ^9^ Department of Pathogen Biology, Base for International Science and Technology Cooperation, Carson Cancer Stem Cell Vaccines R&D Center, International Cancer Center Shenzhen University Medical School Shenzhen China; ^10^ State Key Laboratory of Bioactive Molecules and Drug ability Assessment Jinan University Guangzhou China

**Keywords:** *Enterococcus faecalis*, everolimus, extracellular vesicles, HCC, mTOR, Obg

## Abstract

Hepatocellular carcinoma (HCC) is associated with gut microbiota dysbiosis, yet specific oncogenic mechanisms remain elusive. We identified *Enterococcus faecalis* (EF) as significantly enriched in human liver tumours, where its abundance correlates with disease severity. Both live EF and its conditioned medium promoted HCC cell proliferation, protein translation and tumourigenesis. Mechanistically, EF‐derived extracellular vesicles (EF‐EVs) deliver the bacterial GTPase Obg to activate the host mTOR pathway and drive tumour progression. EF‐Obg contains a Ras‐like G domain homologous to the mTOR regulator Rheb and binds mTOR via a conserved G1 motif. CRISPR interference‐mediated knockdown of *obg* in EF abolished mTOR activation and tumourigenic capacity in vivo. Clinically, high EF‐Obg expression in HCC tissues correlates with mTOR hyperactivation and reduced patient survival. The mTOR inhibitor Everolimus effectively suppressed tumour growth in an EF‐colonied orthotopic model, highlighting its therapeutic potential for HCC patients with high EF burden. Collectively, this work establishes a causal link between tumour‐resident *E. faecalis* and hepatocarcinogenesis, revealing EF‐Obg as a cross‐kingdom activator of mTOR and providing a rationale for microbiota‐guided personalised therapy in HCC.

## Graphical abstract

*E. faecalis* is enriched in HCC tissue and can promote liver tumourigenesis by activating mTOR signalling pathway. Obg derived from *E. faecalis* extracellular vesicles (EVs) is a GTPase physically interacts with mTOR, and acts as an interkingdom activator of mTOR. EF‐Obg expression level in HCC tissue correlates with poor prognosis in HCC patients. EF‐mediated mTOR activation in HCC can be targeted for therapy using mTOR inhibitor everolimus.

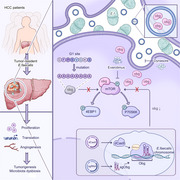

**Abbreviations**
ALB: albumin
ALP: alkaline phosphatase
ALT: alanine aminotransferase
AST: aspartate aminotransferase
BCLC: Barcelona Clinic Liver Cancer
CPZ: chlorpromazine
CytoD: cytochalasin D
DiO: 3,3′‐dioctadecyloxacarbocyanine perchlorates
EF: *Enterococcus faecalis*
EF‐CM: *Enterococcus faecalis* conditioned medium
EF‐dCas‐Obg: *Enterococcus faecalis* dCas9‐Obg knockdown strain
EF‐EVs: *Enterococcus faecalis* extracellular vesicles
EVs: extracellular vesicles
G1 motif: GTP‐binding site 1 motif
GSEA: Gene Set Enrichment Analysis
HUVECs: human umbilical vein endothelial cells
MOI: multiplicity of infection
NES: normalised enrichment score
NTA: nanoparticle tracking analysis
PCoA: principal coordinate analysis
PLA: proximity ligation assay
TBIL: total bilirubin
TEM: transmission electron microscopy
TMA: tissue microarray
TNT: transcription and translation

## 1 Introduction

Liver cancer ranks among the top three causes of cancer‐related mortality worldwide (Siegel et al. 2024). Growing evidence indicates that the gut microbiome plays a critical role in cancer initiation and progression, including in hepatocellular carcinoma (HCC). Through the gut‐liver axis, factors such as intestinal barrier disruption, altered microbial metabolites and chronic hepatic inflammation are linked to HCC development. Additionally, tumour tissues harbour a distinct resident microbiome, with emerging cohort studies suggesting that tissue‐resident microbiota correlate with cancer risk (Zeng et al. 2024), pathological subtype (Buchta Rosean et al. 2019; Pushalkar et al. 2018; Nejman et al. 2020; Dohlman et al. 2021; Hewlett et al. 2018), prognosis (Riquelme et al. 2019) and treatment response (Nejman et al. 2020; Geller et al. 2017; Yu et al. 2017). However, the molecular mechanisms by which tumour‐resident microbes interact with host cells to drive liver cancer progression remain poorly understood.

*Enterococcus faecalis*, a Gram‐positive bacterium, is a major cause of hospital‐acquired infections (Iannetta et al. 2021). It is enriched in the gut microbiota of patients with hepatitis C virus‐related chronic liver disease (Iida et al. 2021) and secretes exotoxins that induce hepatocyte death and liver injury (Duan et al. 2019). GelE‐positive *E. faecalis* has been shown to promote liver tumourigenesis via TLR4‐MyD88 signalling (Iida et al. 2021). While these studies highlight a pathogenic role for gut‐derived *E. faecalis*, its presence and function within the tumour microenvironment are not well characterised. We identified *E. faecalis* as a bona fide tumour‐resident bacterium in HCC, yet how it influences tumour biology or the local microenvironment remains unclear. Bacterial extracellular vesicles (EVs) are important mediators of intra‐ and inter‐species communication, carrying diverse cargo such as proteins, nucleic acids and lipids (Liang et al. 2022; Erttmann et al. 2022; Hong et al. 2023). Tumour‐resident *E. faecalis* may employ EVs to modulate liver cancer cell behaviour, but the composition and functional impact of EF‐EVs in HCC have not been defined. A mechanistic investigation, such as EV proteomics, could reveal functional proteins that underpin how these vesicles promote tumour progression.

The mammalian target of rapamycin (mTOR) is a central regulator of cell growth that integrates signals from nutrients, energy and growth factors to control transcription, translation, metabolism and proliferation (Heberle et al. 2015). mTOR is hyperactivated in 40%–50% of HCC cases, where it drives metabolic reprogramming (Sahin et al. 2004; Schumacher et al. 2005; Sieghart et al. 2007; Semela et al. 2007; Tenen et al. 2021), highlighting the need to elucidate its activation mechanisms in HCC progression (Mei et al. 2024). Canonically, mTOR complex 1 (mTORC1) is activated by growth factors through PI3K/AKT and MAPK signalling, leading to TSC1/2 inhibition and subsequent activation of the small GTPase Rheb. Rheb then stimulates lysosomal mTORC1 and downstream effectors such as S6K and 4EBP1 (Martin et al. 2014; Inoki et al. 2003). Notably, Rheb overexpression can bypass nutrient‐sensing pathways to directly activate mTORC1 (Manning and Cantley 2003), and its yeast homolog mediates cellular adaptation to nutrient availability (Armijo et al. 2016). It remains unknown whether tumour‐resident bacteria possess analogous GTPases or other signalling molecules capable of similarly hijacking the mTORC1 pathway in cancer cells to promote growth.

In this study, we identify *E. faecalis* as a tumour‐resident bacterium that promotes HCC progression. We show that EF‐EVs deliver the bacterial GTPase Obg (EF‐Obg), which shares structural homology with the critical G1 site of Rheb, to activate the host mTOR pathway. CRISPR interference‐mediated knockdown of obg in *E. faecalis* abolishes its oncogenic effects. Clinically, high EF‐Obg expression in HCC tissues correlates with mTOR hyperactivation and poor patient survival. Importantly, the mTOR inhibitor everolimus significantly suppresses tumour growth in orthotopic liver cancer models colonised by *E. faecalis*. Together, our findings establish a causal link between tumour‐resident *E. faecalis* and hepatocarcinogenesis, revealing EF‐Obg as a previously unidentified cross‐kingdom activator of mTOR. This work provides diagnostic insights for *E. faecalis*‐associated HCC and highlights the therapeutic potential of targeting the EF‐mTOR axis in this patient subset.

## 2 Materials and Methods

### 2.1 Key Reagent and Resource

Key reagents and resources are listed in Table S2.

### 2.2 Patient and Public Involvement Statement

Forty‐one patients pathologically diagnosed with primary hepatocellular carcinoma (HCC) who underwent radical hepatectomy at Sun Yat‐sen University Cancer Center (SYSUCC) between December 2023 and July 2024 were included, with the following criteria: Child‐Pugh Class A/B liver function (excluding severe liver failure), BCLC stage A/B (surgically eligible), and age over 18 years; exclusion applied to those with metastatic liver cancer, other malignancies, or use of antibiotics/probiotics or radiotherapy/chemotherapy in the past 3 months.

One hundred patients diagnosed with hepatocellular carcinoma who were treated with radical hepatectomy at Sun Yat‐sen University Cancer Center (SYSUCC) from March 2014 to August 2017 were included. Patients were eligible for the study if they received radical hepatectomy and the samples contained matched tumours and corresponding normal liver tissue. Patients who met the following criteria were excluded: received previous therapies before surgery; received palliative surgery; were pathologically diagnosed with intrahepatic cholangiocarcinoma (ICC), lymphoma or other malignant tumours or serious medical diseases; had incomplete medical records or follow‐up data.

Clinical data were collected from raw case reports and all of the tumours were staged according to the 8th edition of the American Joint Committee on Cancer (AJCC) TNM staging system and the Barcelona Clinic Liver Cancer (BCLC) staging system. The patients were generally followed up every 3 months in the first 2 years and then every 6 months until recurrence appeared in the following 3–5 years. If there was still no recurrence, the patients were followed up once a year. The last follow‐up date was 31 July 2024. The overall survival (OS) was defined as the time from the operation date to the death and the recurrence‐free survival (RFS) was defined as the time from the operation date to recurrence, or metastasis, or the last follow‐up date.

### 2.3 Bacteria Culture, Conditioned Medium Preparation and EVs Isolation

*E. faecalis* strain ATCC‐29212 was obtained from the China General Microbiological Culture Collection Center. *E. faecalis* strain OG1RF was gifted from Prof Xue Liu's lab. They were proliferated in brain heart infusion (BHI) medium or BHI agar under aerobic conditions at 37°C for the rest of the experiments. *E. coli* was grown in Luria–Bertani broth Miller at 37°C with shaking at 200 rpm.

For the conditioned medium preparation, a single *E. faecalis* colony was inoculated into 5 mL of BHI broth for 12 h to reach the late exponential phase of growth with OD600 nm–0.8. After centrifugation at 10,000 *g* for 10 min, the supernatant was filtered through Millex‐GP Filter Unit (0.22 µm pore size, Millipore). The supernatant after filtration was temporarily stored at 4°C for further usage (Xu et al. 2023).

For the EVs isolation, a single *E. faecalis* colony was inoculated into 5 mL of BHI broth and grown overnight. The resulting overnight culture was diluted 1:25 with fresh BHI and grown for 4 h to reach late exponential phase of growth with OD600 nm–0.8. After centrifugation at 10,000 *g* for 20 min, the supernatant was filtered through Millex‐GP Filter Unit (0.22 µm pore size, Millipore). The filtrate was then centrifuged using an ultracentrifugation (Beckman Coulter, USA) at 200,000 *g* for 2 h at 4°C. The supernatant was discarded, and washed with PBS. The final pellets were suspended with 200 µL PBS and temporarily stored at 4°C for further usage. EV protein concentration was measured using the BCA method and the particle size and concentration were analyzed by Nanoparticle tracking analysis (NTA).

For the EVs labelling, *E. faecalis* EVs were labelled with 3,3′‐dioctadecyloxacarbocyanine perchlorates (DiO, MCE, HY‐D0969, 20 µg/mL) for 40 min at 37°C. The supernatant containing unbound DiO was dia‐filtered against PBS buffer using 30 kDa Amicon Ultra centrifugal filters (Merck Millipore, UFC203024) to remove the unbound DiO dye by centrifugation at 4000 *g* for 45 min. And protein concentrations were determined. To examine EVs uptake, MHCC‐97 h grown in 12‐well chamber slides were incubated with DiO‐labelled EVs or the sham control for different time periods. The slides were then washed with PBS and fixed with 4% PFA for 15 min at room temperature. After washing with PBS, the slides were stained with DAPI for 15 min and imaged with confocal microscopy using a 40× oil immersion objective lens. Fluorescence quantification was performed using ImageJ software.

For the EVs tracing in vivo, EVs were labelled with Cy7 (MCE, HY‐D0825) for fluorescent imaging. Mice were oral gavaged with Cy7‐labelled EVs and were recorded fluorescence after 1.5 h by IVIS Spectrum In Vivo Imaging System. The mice were sacrificed and harvested livers to analyse fluorescent signals.

### 2.4 Obg Overexpression in *E. coli*

Obg cDNA was amplified by PCR from *E. faecalis* cells. The products were digested with HindIII and BamHI restriction enzymes, ligated into a PET21a vector using T4 DNA ligase, and transformed separately in DH5a cells of *E. coli* for cloning. Colonies on ampicillin agar plates were screened for successful construct formation by colony PCR and double restriction digestion. The recombinant constructs were transformed into BL21 (DE3) competent cells of *E. coli*, and protein expression was induced with 1 mM isopropyl β‐D‐1‐thiogalactopyranoside (IPTG) at 18°C for 16 h. The cells were isolated by centrifugation (6000 *g*, 4°C), dissolved in lysis buffer (50 mM Tris–HCl pH 7.5, 1 mM EDTA, 150 mM NaCl, 0.1% NP‐40, 0.1% Triton‐100, 0.01% SDS) containing protease inhibitors cocktail and phosphatase inhibitors (Bimake), and disrupted by sonication. The supernatant‐containing proteins were collected after centrifugation (12,000 *g*, 4°C, 30 min). The primers were listed in the Table S3.

### 2.5 Obg Knockdown by CRISPR Interference System

The CRISPR interference was performed as previously described. We used *E. faecalis* strain OG1RF, which is more suitable for electroporation. Briefly, two plasmids were used: pMSP3545‐dCas9str (Addgene, #153516) encoding dCas9, and pGCP123‐sgRNA encoding sgRNA. The sgRNA targeting the gene *obg* was designed on the CHOPCHOP website (https://chopchop.cbu.uib.no/). The sequence was synthesised by BGI (The Beijing Genomics Institute) and cloned into plasmid pGCP123‐EbpA‐g1 (Addgene, #153517) with restriction sites PstI and BamHI, and ligated with T4 DNA ligase. This plasmid was subsequently transformed into DH5a. The sgRNA target obg sequence is listed in Table S4.

We followed the procedure provided by Dr. Kimberly A. Kline. Briefly, *E. faecalis* dilute culture 1:10 into prewarmed fresh BHI (100 mL) and incubate until culture OD at 600 nm reaches 0.5–0.75. Then chill cells on ice and pellet cells at 6000 rpm for 10 min at 4°C. Resuspend pellet in 2 mL ice cold sterile H_2_O and split into two 1.5 mL tubes. Pellet at 13,000 rpm for 1 min. Resuspend each pellet in 500 µL lysozyme solution (10 mM Tris pH 8.0, 20% sucrose, 10 mM EDTA, 50 mM NaCl) containing 30 µg/mL lysozyme. Incubated at 37°C for 20 min. Wash each pellet four times with 0.5 mL ice‐cold electroporation buffer (0.5 M Sucrose, 10% glycerol). Resuspend each tube in a total of 300 µL EB/tube and split into 50–65 µL aliquots. Use fresh or store at –80°C.

Electroporation was performed in 0.2‐cm‐gap electroporation cuvettes (Bio‐Rad) at 25 µF, 400 Ω and 2.5 kV. After electroporation, 1 mL SBHI medium, containing 0.5 M sucrose in BHI medium, was immediately added to transformed cells, and the cells were left to recover for at least 2 h at 37°C without shaking. The recovered cells were then plated on selective BHI agar plates. Erythromycin (2 µg/mL) was used to maintain the pMSP3545 plasmid in *E. faecalis*, and kanamycin (500 µg/mL) was used to maintain pGCP123 and its derivatives. We induced the target gene expression with 25 ng/mL nisin (MCE, HY‐P1607). The inhibition efficiency of Obg was evaluated by qPCR.

### 2.6 Preparation of Obg Antibody

The anti‐Obg antibody was prepared by SinoBiological Confidential in Peking. 5 Balb/c mice were immunised with each of two VLP‐conjugated peptides (i.e., 5 mice per peptide). After four immunisations with the protein and three with the peptides, ELISA binding assays confirmed that all 10 mice met the fusion criteria for immunogenicity. After 1 hybridoma fusion, a total of two strains of hybridoma cell strains were obtained that bound to both antigenic proteins and clinical samples provided by the client. Subsequently, four strains of mouse monoclonal antibody were purified to meet the required quantity and achieved purity exceeding 90%. These antibodies were validated to bind to the antigen proteins in ELISA assays.

### 2.7 Statistical Analysis

Mean ± SD (at least three biological replicates or three independent experiments) was used to plot the data. Student's *t*‐test, one‐way Analysis of Variance (ANOVA) test, and two‐way ANOVA test were used to analyse quantitative data between groups. Kaplan‒Meier curves were generated to estimate OS, RFS and differences between curves were evaluated using a log rank test. A value of *p* < 0.05 was considered statistically significant.

## 3 Results

### 3.1 *Enterococcus faecalis* Is Enriched in HCC Tissues and Promotes the Proliferation and Migration of Cancer Cells

**FIGURE 1:**
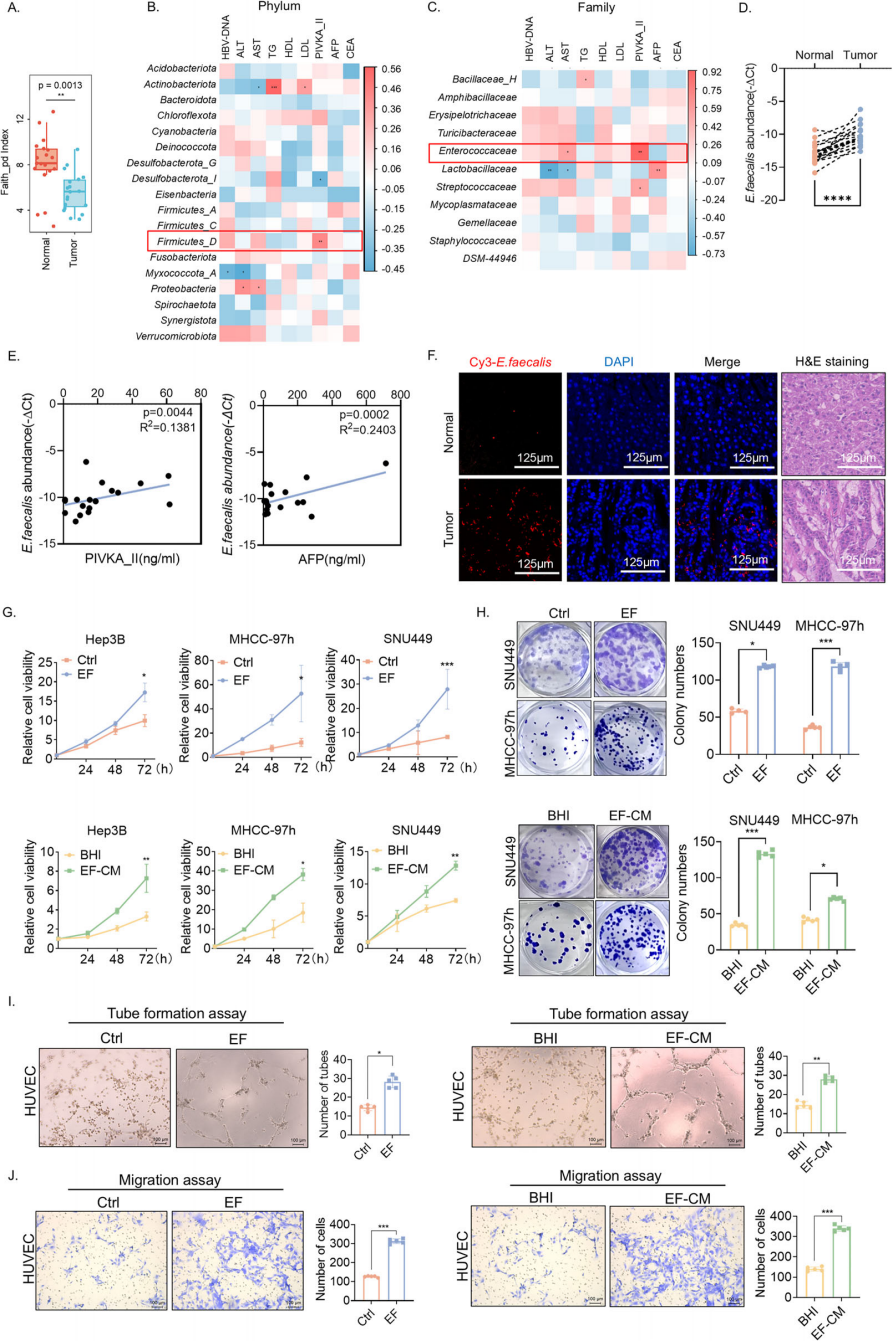
*E. faecalis* is enriched in HCC tissues and promotes the proliferation of liver cancer cells. (A) Faith_pd index showed the alpha diversity of microbiota extracted from liver tumour tissues and normal tissues (*n* = 19). (B and C) Spearman's correlations between the relative abundance of bacterium and clinical parameters of HCC patients. The red box indicates the Phylum and Family of *E. faecalis*. (D) Relative *E. faecalis* abundance in tissues detected by qPCR. (E) Spearman's correlation between *E. faecalis* abundance and serum PIVKA_II or AFP level in HCC patients. (F) Detection of *E. faecalis* in tissues by fluorescence in situ hybridisation (FISH). Sections of tissue were counterstained with DAPI (blue) and *E. faecalis* probes (red). H&E staining was shown. Scale bar = 125 µm. (G and H) CCK8 assay and Colony formation assay of the indicated HCC cells treated with EF or EF‐CM were shown. (I and J) Tube formation and migration assay of HUVEC cells were shown. Scale bar = 100 µm. The Wilcoxon test was used to calculate the *p* value between two groups (A). Unpaired two‐tailed Student's *t* test was used in (B), (C), (G), (H), (I) and (J). Paired *t*‐test was used in (D).

To investigate the contribution of hepatic tissue‐colonising microbes to the progression of hepatocellular carcinoma (HCC), we profiled the microbiota of tumour tissues and matched para‐cancerous tissues using 16S rRNA sequencing. While principal coordinate analysis (PCoA) revealed no significant compositional differences between groups (Figure S1A,B), alpha diversity was significantly reduced in tumour tissues (Figure 1A). Notably, the abundance of *Firmicutes_D* showed a positive correlation with the HCC biomarker des‐gamma‐carboxyprothrombin (PIVKA‐II), and *Enterococcaceae* (a family within *Firmicutes_D*) correlated positively with both PIVKA‐II and aspartate aminotransferase (AST) levels (Figure 1B,C). These associations suggest that *Enterococcaceae* may play a role in promoting hepatocarcinogenesis.

To further define the specific Enterococcaceae species associated with HCC, we quantified several well‐studied members—*E. casseliflavus*, *E. faecium*, *E. faecalis* and *E. mundtii*—in tissue samples (*n* = 40) using real‐time PCR. *E. faecium* and *E. mundtii* were nearly undetectable in all samples. *E. faecalis* was significantly enriched in tumour tissues compared with adjacent normal tissues (Figure 1D), whereas *E. casseliflavus* abundance did not differ (Figure S1C). Correlation analysis further demonstrated that *E. faecalis* abundance, but not that of *E. casseliflavus*, was significantly correlated with levels of the HCC biomarkers PIVKA‐II and AFP (Figures 1E and S1D). Finally, fluorescence in situ hybridization (FISH) confirmed the presence of tumour‐resident *E. faecalis* (Figure 1F). Besides, we divided the patients into EF‐detectable group and EF‐undetectable group based on qPCR result. The Kaplan‒Meier survival curve showed that patients in the EF‐detectable group had shorter recurrence‐free survival (RFS) than those in the EF‐undetectable group (Figure S1E). Since the patients in our cohort are all early‐stage patients eligible for surgical treatment, there has been no difference in overall survival so far. For the limitation of insufficient sample size in our own cohort, we further verified the correlation between *Enterococcus* abundance and HCC prognosis using TCGA‐LIHC databases combined with tissue flora annotation information (Chen et al. 2023). The results showed a trend toward an association between high *Enterococcus* abundance and poor overall survival (OS) (*p* = 0.064) (Figure S1F). In particular, high *Enterococcus* abundance was significantly correlated with poor overall survival (OS) in HBV‐positive patients (*p* = 0.012) (Figure S1G). Together, these results indicate that *E. faecalis* is a tumour‐enriched microbe whose presence correlates with key clinical parameters of HCC.

To evaluate the oncogenic potential of *E. faecalis* in HCC, we co‐cultured HCC cell lines with the bacterium at a multiplicity of infection (MOI) of 30. CCK‐8 and colony formation assays revealed that *E. faecalis* significantly promoted HCC cell proliferation and colony formation (Figure 1G,H). In parallel, *E. faecalis* co‐culture enhanced tube formation and migration of human umbilical vein endothelial cells (HUVECs) (Figure 1I,J). Given that bacterial metabolites and secreted proteins often mediate such effects (Xu et al. 2023), we prepared a conditioned medium from *E. faecalis* cultures (referred to hereafter as EF‐CM) and tested its impact on cell growth and migration. Consistent with the live‐bacteria results, EF‐CM also robustly promoted HCC cell proliferation as well as HUVECs tube formation and migration (Figure 1G–J). Collectively, these data demonstrate that both live *E. faecalis* and its secreted factors can stimulate HCC cell growth and endothelial cell migration.

### 3.2 *Enterococcus faecalis* Drives HCC Tumourigenesis Through Potentiation of the mTOR Signalling Pathway

**FIGURE 2:**
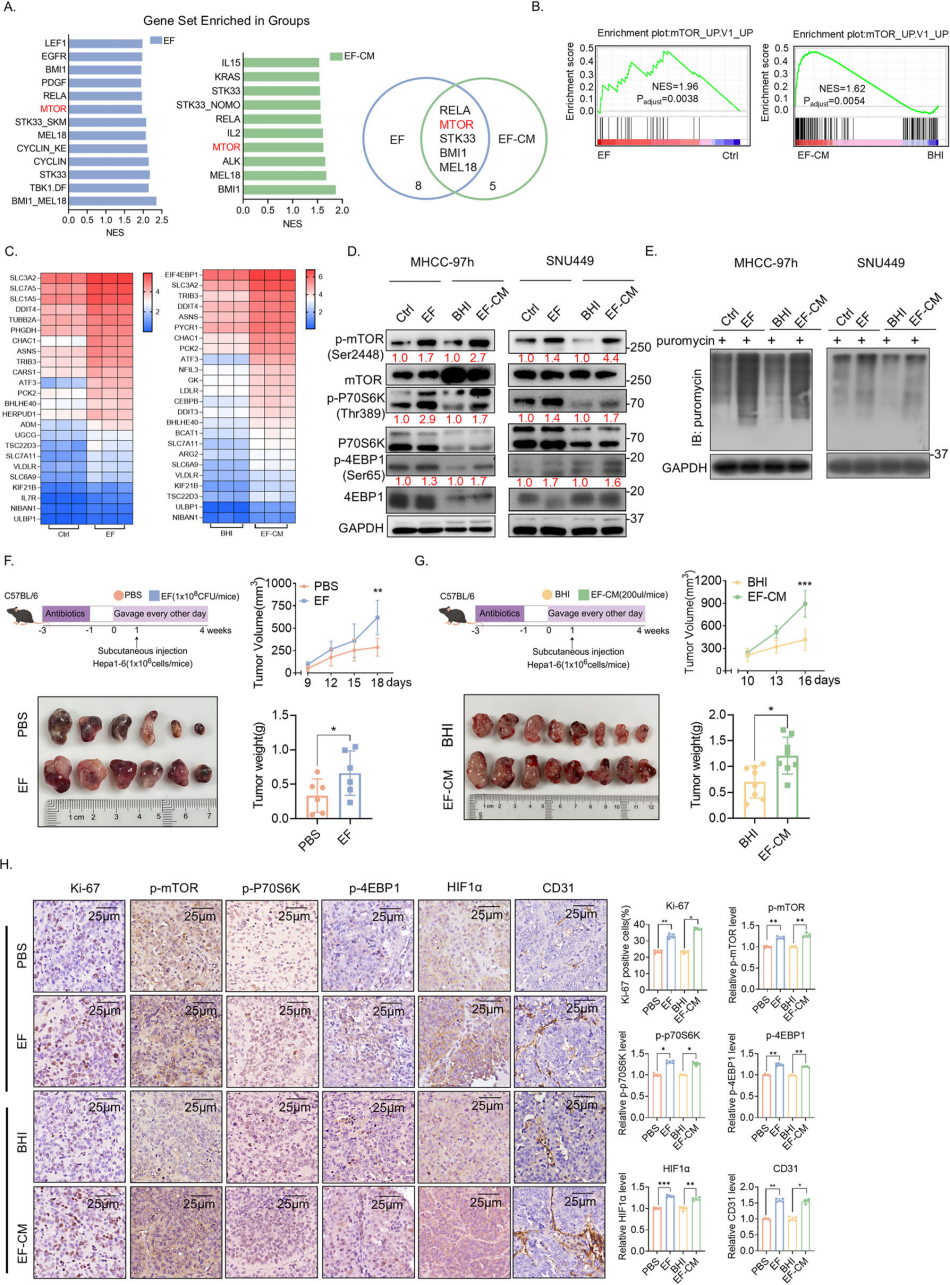
*E. faecalis* enhances mTOR signalling and promotes liver tumourigenesis. (A) Significantly enriched cancer‐related pathways in Hep3B cells after indicated treatment. Overlapping pathways enriched in both EF and EF‐CM treatment (NES > 1.5) were shown. (B) GSEA plots of mTOR signalling pathway correlated with indicated treatment. (C) Heatmap showed mRNA levels of mTOR signalling pathway‐related genes after indicated treatments, as revealed by RNA‐sequencing analysis. (D) Protein levels of mTOR, P70S6K, 4EBP1 and their phosphorylation levels in HCC cells after indicated treatments. Quantification was shown as red numbers. (E) Anti‐puromycin immunoblot analysis showed translation activities in HCC cells following the indicated treatments. (F and G) Schematic diagram of animal experiments. Tumour volumes, tumour weights and representative tumour pictures were shown (*n* = 6 in F, *n* = 8 in G). (H) Representative images of immunohistochemical staining from tumour samples following the indicated treatments. Quantification of IHC staining was shown as bar graphs (right). Permutation test was used to calculate the *p* value in (A) and (B). Unpaired two‐tailed Student's *t* test was used in (F), (G) and (H). NES, normalised enrichment score.

To elucidate the precise mechanism underlying the effects of *E. faecalis*, we performed RNA sequencing on Hep3B cells following either infection with the bacterium or treatment with its conditioned medium (EF‐CM). Gene Set Enrichment Analysis (GSEA) revealed significant enrichment of several pathways in both treatment groups (Figure 2A). Considering that dysregulation of the PI3K/AKT/mTOR signalling pathway is common in HCC, which makes it a focus of ongoing research and a key target for developing novel liver cancer therapies, we selected the mTOR signalling pathway for further investigation (Figure 2B). Concordantly, a heatmap visualization showed the upregulation of most mTOR downstream target genes under both conditions (Figure 2C). Immunoblotting confirmed that both *E. faecalis* infection and EF‐CM treatment enhanced the phosphorylation of mTOR and its key downstream effectors, p70S6K and 4E‐BP1 (Figure 2D). Furthermore, anti‐puromycin immunoblot analysis demonstrated a corresponding increase in global protein synthesis following these treatments (Figure 2E). Taken together, these results indicate that *E. faecalis* and its secreted factors activate the mTOR signalling pathway, thereby enhancing protein synthesis.

To further characterise the functional role of *E. faecalis* in HCC development in vivo, we established a subcutaneous allograft model in C57BL/6 mice using the mouse liver cancer cell line Hepa1‐6. Mice were administered *E. faecalis* or its conditioned medium (EF‐CM) intragastrically every other day, with PBS or BHI medium serving as the respective vehicle controls. Compared to controls, both *E. faecalis* and EF‐CM administration promoted tumour growth, resulting in significantly increased tumour volumes, greater tumour weights and a denser intratumoural vascular network (Figure 2F,G). Immunohistochemical analysis revealed a significant increase in Ki‐67‐positive cells within tumours from both treatment groups. Consistent with our in vitro findings, protein levels of phosphorylated mTOR and its downstream effectors (p‐p70S6K, p‐4E‐BP1), as well as the markers HIF‐1α and CD31, were also elevated in these tumours (Figure 2H). Collectively, these results demonstrate that *E. faecalis* promotes HCC progression in vivo by activating the mTOR signalling pathway.

### 3.3 *E. faecalis* EVs, Delivered by Dynamin‐Dependent Endocytosis, Activate the mTOR Pathway to Promote Liver Cancer Cell Proliferation

**FIGURE 3:**
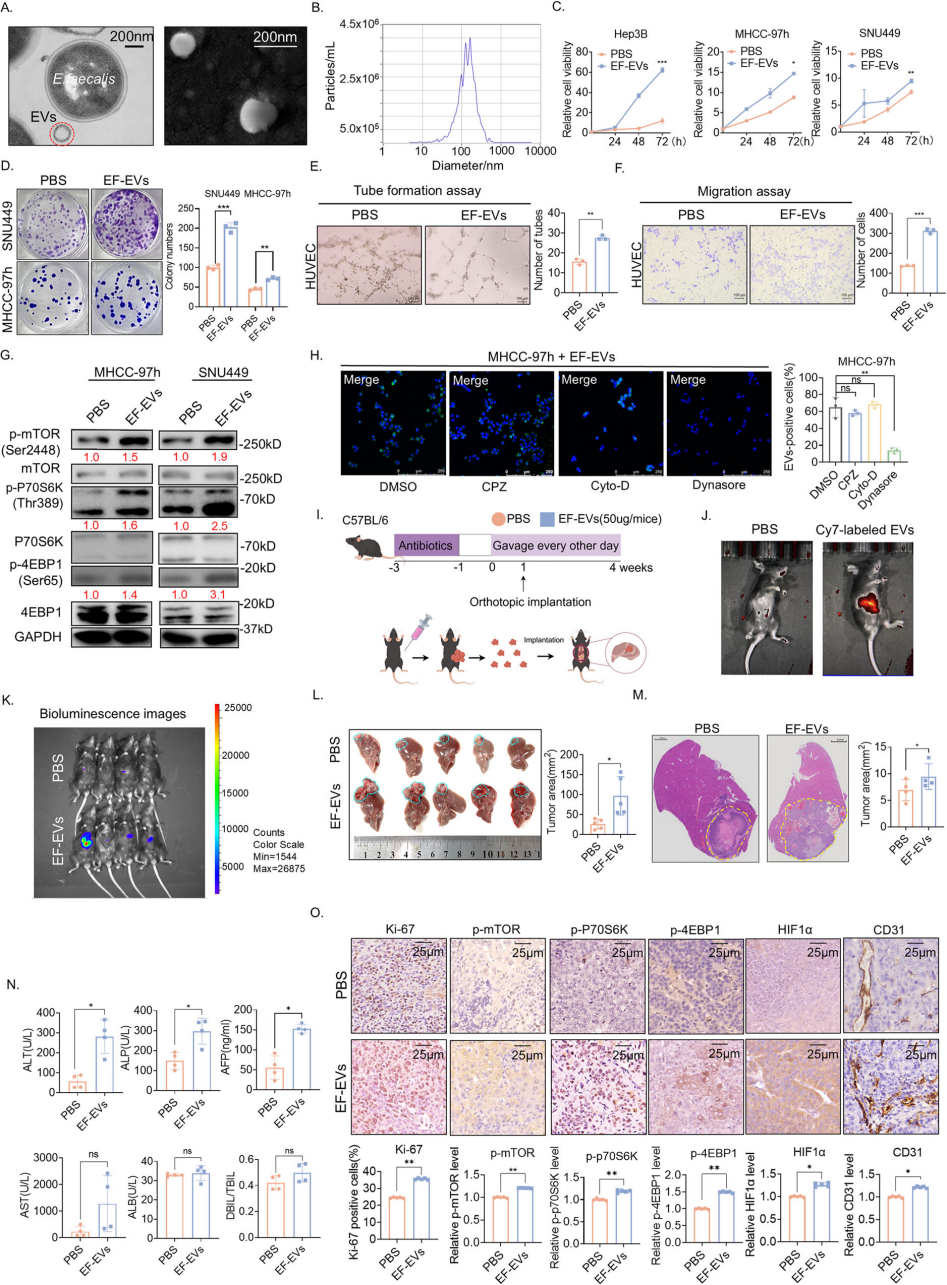
*E. faecalis* EVs promote liver cancer progression and activate mTOR in vitro and in vivo. (A) Transmission electron microscopy (TEM) image of *E. faecalis* (cross‐section) with secreted EVs (red circle). Scale bar = 200 nm. (B) Representative TEM image and nanoparticle tracking analysis (NTA) of EF‐EVs. (C and D) CCK8 assay and Colony formation assay of the indicated HCC cells treated with EF‐EVs were shown. (E and F) Tube formation and migration assay of HUVEC cells under the indicated treatments were shown. Scale bar = 100 µm. (G) Protein levels of phosphorylation of mTOR, P70S6K and 4EBP1 in HCC cells under the indicated treatments. Quantification was shown as red numbers. (H) Confocal micrographs of MHCC‐97 h cells pretreated with indicated inhibitors (Chlorpromazine 5 µM, Cytochalasin D 2.5 µM, Dynasore 40 µM) for 1 h, followed by incubation with 20 µg/mL DiO‐labelled EVs for 120 min. Scale bars = 250 µm. Quantification of EVs‐positive cells was shown as bar graphs. (I) Scheme of the EF‐EVs‐mediated liver tumourigenesis experiment. (J) Representative fluorescence images of mice 90 min after gavage with Cy7‐labelled EF‐EVs (right) or PBS (left). (K) Bioluminescence images of the mouse's whole body at the end of the experiments. (L) Images of livers harvested from EF‐EVs (*n* = 5) and the PBS (*n* = 5) group. The blue dashed lines indicate the tumour borders. (M) Representative images of H&E staining on liver tissue sections (left). (N) Quantification of serum ALT, ALP, AFP, AST, ALB levels and the rate of DBIL/TBIL from two groups. (O) Representative images of immunohistochemical staining in tumour samples from the indicated treatments. Scale bar = 25 µm. Unpaired two‐tailed Student's *t* test was used in (C), (D), (E), (F), (H), (L), (M), (N) and (O). EF‐EVs, *E. faecalis* extracellular vesicles; DIO, 3,3'‐dioctadecyloxacarbocyanine perchlorates; DMSO, dimethyl sulfoxide; CPZ, chlorpromazine; CytoD, cytochalasin D. ALT, alanine transaminase; ALP, alkaline phosphatase; AFP, α‐fetoprotein; AST, aspartate aminotransferase; ALB, albumin; DBIL, direct bilirubin; TBIL, total bilirubin.

Bacterial extracellular vesicles (EVs) are key mediators of bacterial‐host interactions. Transmission electron microscopy (TEM) revealed numerous spherical EVs on the surface of *E. faecalis* (Figure 3A). Scanning electron microscopy and nanoparticle tracking analysis (NTA) confirmed the isolation of EVs as spherical, bilayer membrane‐enclosed vesicles with a mean diameter of approximately 160 nm (Figure 3B), consistent with the characteristic features of Gram‐positive bacterial EVs (Briaud and Carroll 2020). Treatment of HCC cells with *E. faecalis* EVs (EF‐EVs) at a concentration of 50 µg/mL significantly promoted cell proliferation, as demonstrated by CCK‐8 and colony formation assays (Figure 3C,D). Furthermore, EF‐EVs also enhanced tube formation and migration of human umbilical vein endothelial cells (HUVECs) (Figure 3E,F). Immunoblotting confirmed that EF‐EV treatment activated the mTOR signalling pathway in HCC cells, evidenced by increased phosphorylation of mTOR and its downstream targets p70S6K and 4E‐BP1 (Figure 3G). These results identify EF‐EVs as functional effectors that mediate mTOR pathway activity in HCC.

EVs, which are enclosed by a lipid bilayer, are typically internalised by cells via endocytosis. To investigate the cellular uptake of *E. faecalis* EVs (EF‐EVs) by HCC cells, we labelled EF‐EVs with the lipophilic membrane dye 3,3′‐dioctadecyloxacarbocyanine perchlorate (DiO), which emits green fluorescence upon integration into the cell membrane (Wang et al. 2020). Incubation of MHCC‐97H cells, 293T cells and THLE‐2 cells with DiO‐labelled EF‐EVs for 2 h resulted in distinct intracellular green fluorescence, confirming EVs uptake. Additionally, MHCC‐97 h cells exhibited the highest uptake of EF‐EVs, followed by 293T cells with relatively lower uptake, while the THLE‐2 cells displayed the least uptake (Figure S3A). This indicated that HCC cells may preferentially take up EF‐EVs due to activated endocytosis. To delineate the specific endocytic pathway involved, we pretreated MHCC‐97 h cells with pharmacological inhibitors targeting distinct mechanisms: chlorpromazine (clathrin‐mediated endocytosis), dynasore (dynamin‐dependent endocytosis) and cytochalasin D (actin polymerization‐dependent endocytosis). Immunofluorescence analysis revealed that dynasore significantly reduced EF‐EVs internalisation, whereas chlorpromazine and cytochalasin D had no significant effect (Figure 3H). These results demonstrate that EF‐EVs enter HCC cells primarily through a dynamin‐dependent endocytic pathway to mediate their biological effects.

### 3.4 EF‐EVs Drive Hepatocarcinogenesis and Associated Pathology in Mice by Amplifying mTOR Signalling

To validate the in vivo effects of *E. faecalis* EVs (EF‐EVs), we established an orthotopic liver cancer model in C57BL/6 mice as previously described (Fan et al. 2012). Following a 2‐week antibiotic pretreatment, mice were randomly divided into two groups and administered EF‐EVs or PBS via oral gavage every other day until the experimental endpoint (Figure 3I). For in vivo tracking, fluorescence imaging at 90 min post‐gavage revealed that Cy7‐labelled EF‐EVs had distributed to the liver (Figure 3J). Administration of EF‐EVs significantly promoted HCC progression, resulting in stronger bioluminescence signals and larger orthotopic tumour volumes compared to the PBS‐treated group (Figure 3K–M). Moreover, serum levels of the liver damage markers alanine aminotransferase (ALT), aspartate aminotransferase (AST) and alkaline phosphatase (ALP) were elevated following EF‐EV treatment (Figure 3N). Immunohistochemical analysis further showed increased levels of phospho‐mTOR, phospho‐p70S6K, phospho‐4E‐BP1, HIF‐1α and CD31, as well as a higher Ki‐67 index, in tumour tissues from EF‐EV‐treated mice (Figure 3O). These results demonstrate that EF‐EVs promote HCC progression *in vivo* by activating the mTOR signalling pathway.

16S rRNA profiling of fecal samples revealed that EF‐EV administration significantly reduced the overall abundance of the gut microbiota (Figure S2A) and altered its composition (Figure S2B), indicating a shift in the relative proportions of different bacterial taxa (Figure S2C, D). Notably, EF‐EV treatment reduced the colonisation of beneficial probiotics, including *Akkermansia*, butyrate‐producing *Clostridiales*, and *Bifidobacterium*, while promoting the expansion of *E. faecalis* itself as well as potentially harmful genera such as *Fusobacterium*, *Streptococcus*, and *Veillonella* (Figure S2E,F). These results suggest that EF‐EVs can remodel the gut microbial ecosystem by depleting health‐associated taxa and enriching for opportunistic or pathogenic bacteria. Such dysbiosis may impair gut health and host immune regulation, thereby creating a permissive environment for liver tumourigenesis.

### 3.5 Obg, a GTPase Derived From *E. faecalis* EVs, Physically Interacts With mTOR

**FIGURE 4:**
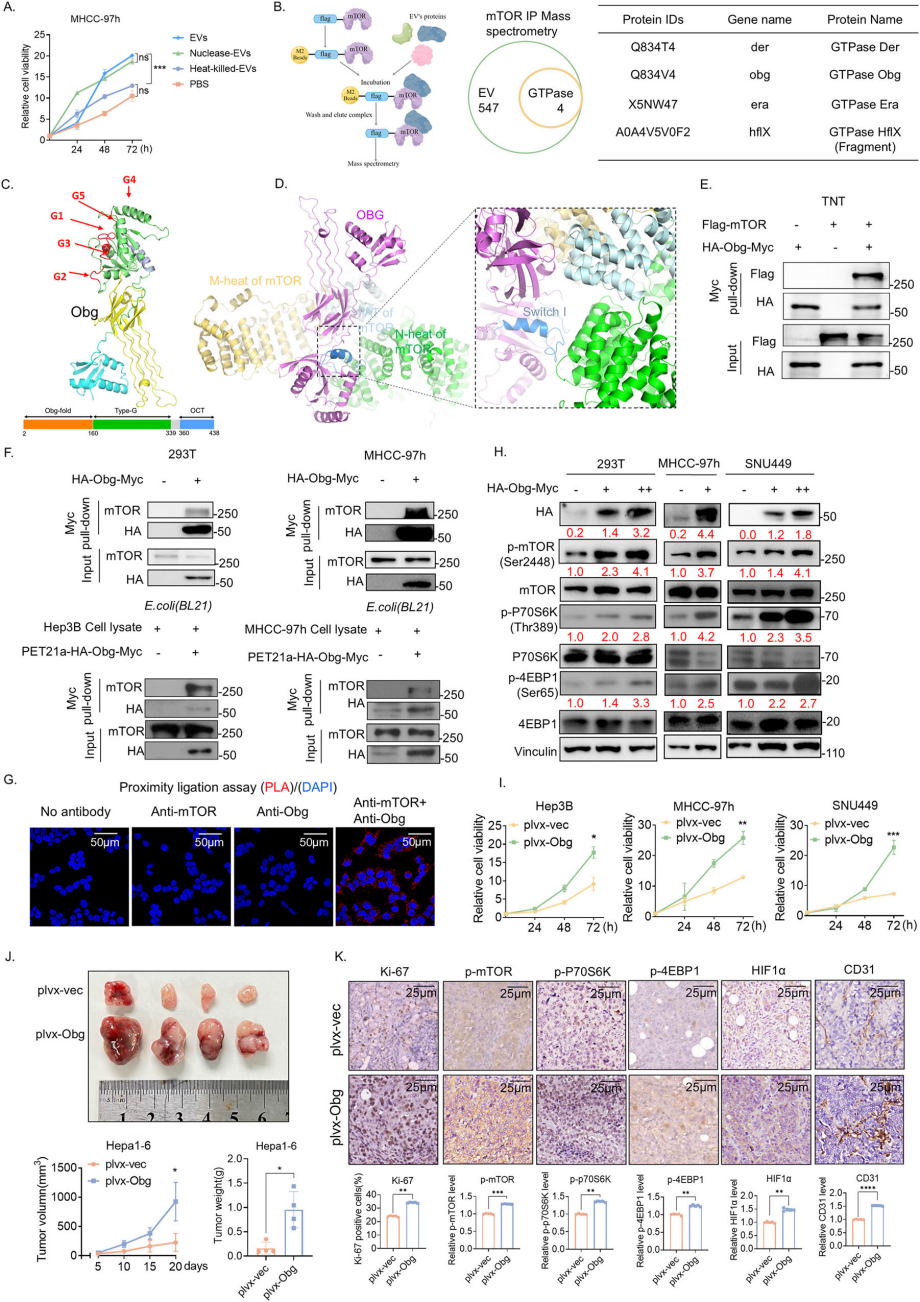
EF‐Obg, a GTPase derived from *E. faecalis* extracellular vesicles (EVs), physically interacts with mTOR. (A) The growth curves of MHCC97h cells with indicated treatments were measured by CCK8 assay. (B) Schematic diagram of identification of mTOR‐bound EF‐EV proteins. Four GTPases from EF‐EV were identified from the mTOR IP Mass spectrometry (right). (C and D) GTPase‐Obg protein structure with domains (C) and Obg‐mTOR interaction structure (D) visualised by PyMOL. The red arrows showed the GTP binding sites (G1‐G5). (E) In vitro co‐IP assays were performed. The protein products were obtained from transcription and translation (TNT) assays. (F) Co‐IP assays were performed using exogenously expressed HA‐Obg‐Myc in 293T/MHCC‐97H cells, or using bacterially expressed (from *E. coli BL21* cells) PET‐21a‐HA‐Obg‐Myc mixed with Hep3B/MHCC‐97H cell lysates. (G) Representative image results of proximity ligation assay (PLA) using antibodies against mTOR and Obg. The red signals indicate interactions between mTOR and Obg. DAPI was used to stain the cell nuclei (blue signals). Scale bar = 50 µm. (H) Protein levels of phosphorylation of mTOR, P70S6K and 4EBP1 after HA‐Obg overexpression in indicated cells. Quantification was shown as red numbers. (I) Cell growth curves of indicated HCC cells overexpressing Obg. Cell growth was measured by CCK8 assay. (J) C57BL/6 female mice were randomly grouped and subcutaneously injected with 5 × 10^5^ Hepa1‐6 cells overexpressing Obg (plvx‐Obg) or control (plvx‐vec). Representative tumour picture, tumour volumes and tumour weights were shown (*n* = 4). (K) Representative images of immunohistochemical staining from tumour samples of indicated group. Scale bar = 25 µm. One way ANOVA test was used in (A). Unpaired two‐tailed Student's *t* test was used in (I), (J) and (K).

Bacteria‐derived proteins, DNA and RNA can interact with host cell molecules to modulate key signalling pathways and influence tumour progression (Jiang et al. 2023; Zhang et al. 2025). To identify the active component of *E. faecalis* EVs (EF‐EVs), we treated MHCC‐97H cells with EF‐EVs pretreated with either universal nuclease (to degrade nucleic acids) or heat (to denature proteins). Heat‐killed EVs lost their ability to promote HCC cell proliferation, whereas nuclease‐treated EVs remained active (Figure 4A), indicating that protein components mediate the oncogenic effect. Mass spectrometry analysis of EF‐EV protein content revealed a significant enrichment of ribosome‐associated proteins involved in translation (Figure S3B,C). To identify specific EV proteins that interact with host signalling machinery, we performed a co‐immunoprecipitation (co‐IP) mass‐spectrometry screen. FLAG‐tagged mTOR was overexpressed in HEK293T cells and used to pull down interacting proteins from EF‐EV lysates. This screen identified 547 EF‐EVs‐derived proteins/peptides (Figure 4B). Given that the Ras‐family GTPase Rheb activates lysosomal mTORC1 in mammalian cells, we hypothesised that an EF‐EV GTPase might functionally mimic Rheb to promote HCC growth. Among several GTPases detected, the spo0B‐associated GTP‐binding protein (Obg) was of particular interest. Obg proteins are conserved TRAFAC‐class P‐loop GTPases involved in bacterial DNA replication, ribosome maturation, and stress response (Kint et al. 2014)—functions analogous to those regulated by mTOR in eukaryotes. EF‐Obg contains an N‐terminal domain, a central G‐domain common to all Obg proteins, and a non‐conserved C‐terminal region (Figure 4C). Its G‐domain exhibits a Ras‐like fold with five conserved motifs for GTP/GDP binding and hydrolysis, structurally resembling Rheb (Chakraborty et al. 2022). Structural modelling predicted that EF‐Obg could form a complex with mTOR analogous to the Rheb‐mTOR interaction (Figure 4D). Direct binding was confirmed using an in vitro transcription‐translation (TNT) system (Figure 4E) and further validated by co‐IP in HEK293T and MHCC‐97H cells (Figure 4F). Proximity ligation assay (PLA) additionally demonstrated their interaction in live cells (Figure 4G). Considering that mTOR is localised on lysosomes, we performed dual and multiplex immunohistochemistry. We overexpressed Obg or directly added EF‐EVs to 293T cells, then stained for mTOR, Obg and lysosomal marker LAMP1. The confocal microscopy showed that Obg was co‐localised with LAMP1 and mTOR, indicating that Obg could localise to lysosomes and interact with mTOR (Figure S3D). Taken together, these data establish a direct physical interaction between mTOR and the EVs‐derived GTPase EF‐Obg.

### 3.6 The GTPase Activity of *E. faecalis* Obg Is Critical for mTOR Activation and the Subsequent Promotion of Hepatocarcinogenesis

EF‐Obg binding enhanced mTOR pathway activation in a dose‐dependent manner, confirming its functional role in mTOR regulation (Figure 4H). Consistent with this, overexpression of EF‐Obg promoted HCC cell proliferation, as measured by CCK‐8 assay (Figure 4I). In a subcutaneous mouse model, C57BL/6 mice injected with Hepa1‐6 cells stably overexpressing EF‐Obg developed larger and heavier tumours compared to controls (Figure 4J). Immunohistochemical analysis of these tumours confirmed activation of the mTOR pathway in the EF‐Obg‐expressing group (Figure 4K). These results indicate that EVs‐derived EF‐Obg promotes liver tumourigenesis by activating the mTOR pathway.

**FIGURE 5:**
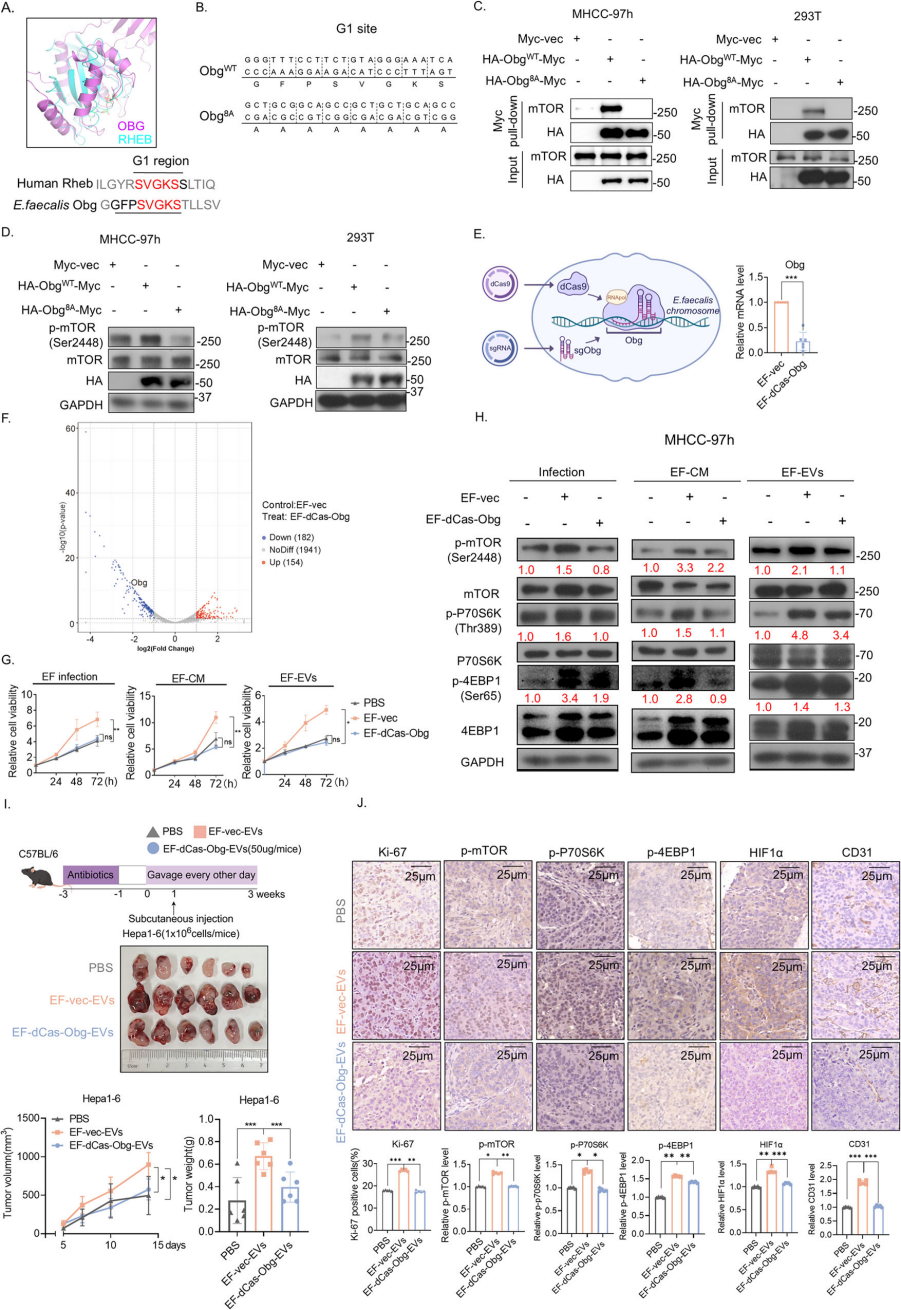
The *obg* gene is critical for *E. faecalis*‐mediated mTOR activation and hepatocarcinogenesis. (A) Comparison of GTP binding sites 1 (G1) homology between Obg and Rheb. Aligned amino acid sequences in G1 were highlighted. (B) The detailed nucleotide sequence and the translated amino acids of the G1 site from EF‐Obg wild‐type (Obg^WT^) and G1 site mutant (Obg^8A^). (C) The interaction between mTOR and Obg^WT^ or Obg^8A^ was determined by co‐IP assay. (D) The phosphorylation levels of mTOR following Obg^WT^ or Obg^8A^ overexpression were shown. (E) Schematic diagram illustrating the knockdown of Obg by CRISPR interference system. And Obg mRNA level was measured by qPCR in *E. faecalis obg* gene knockdown strain (EF‐dCas‐Obg). (F) Volcano blot showed differentially expressed genes in EF‐dCas‐Obg strain compared with EF‐vec strain, as revealed by RNA‐sequencing analysis. The results showed that Obg is downregulated. (G) The growth curves of MHCC‐97 h cells with indicated treatments were measured by CCK8 assays. (H) Phosphorylation and total levels of mTOR, P70S6K, 4EBP1 in HCC cells with indicated treatments were measured by immunoblot assay. Quantification was shown as red numbers (below). (I) Schematic diagram of animal experiments. Representative tumour image, tumour volumes and tumour weights were shown (*n* = 6). (J) Representative images of immunohistochemical staining from tumour samples of the indicated group. Scale bar = 25 µm. Unpaired two‐tailed Student's *t* test was used in (E); One‐way ANOVA was used in (G), (I) and (J). G1 site, GTP‐binding site 1.

Structural comparison of EF‐Obg with the canonical mTOR‐activating GTPase Rheb using PyMOL revealed a conserved amino acid sequence at the G1 motif (Figure 5A). To assess the functional importance of this site, we introduced a missense mutation into the EF‐Obg G1 motif, generating the mutant Obg^8A^(Figure 5B). Co‐immunoprecipitation (co‐IP) experiments demonstrated that the Obg^8A^ mutant lost its ability to bind mTOR (Figure 5C). Accordingly, while wild‐type (WT) EF‐Obg activated mTOR signalling, the Obg^8A^ mutant failed to do so (Figure 5D). To investigate the competitive or dynamic regulation between EF‐Obg and endogenous Rheb, we overexpressed mTOR and Obg in MHCC‐97 h cells. CO‐IP results showed that EF‐Obg^WT^ enhanced the binding of Rheb to mTOR in a dose‐dependent manner. However, the EF‐Obg^8A^ mutant exhibited no promotive effect on the interaction between Rheb and mTOR. We also knocked down *rheb* in MHCC‐97 h cells, the results showed that knockdown *rheb* markedly abolished the activation of the mTOR signalling pathway induced by EF‐Obg (Figure S4A,B). Together, these results indicated that EF‐Obg exerted a synergistic effect with Rheb in activating the mTOR signalling pathway via the G1 domain.

Considering that Obg is a universally conserved GTPase in bacteria, we selected two non‐carcinogenic commensal bacteria, *Escherichia coli* and *Akkermansia muciniphila*. We extracted EVs from these bacteria and overexpressed their Obg homologs. The results showed that EVs from *Escherichia coli* and *Akkermansia muciniphila* failed to activate the mTOR signalling pathway, and neither could their Obg homologs. Then we aligned the sequences of their Obg homologs and found marked differences in the G1 motif (Figure S4C, D, andE). Together, these findings establish the G1 motif as essential for EF‐Obg's interaction with mTOR and its subsequent oncogenic activity.

To define the role of the *E. faecalis obg* gene in mTOR activation and hepatocarcinogenesis, we used a CRISPR interference (CRISPRi) system (Afonina et al. 2020) to generate an *obg* knockdown strain (EF‐dCas‐Obg) (Figure 5E). The sgRNA target sequences for EF‐Obg were designed using CHOPCHOP (Table S4). Successful knockdown was confirmed by qPCR and RNA sequencing (Figure 5E,F). The EF‐dCas‐Obg strain exhibited impaired growth relative to the control, indicating that *obg* is essential for normal *E. faecalis* proliferation (Figure S5A). Nanoparticle tracking analysis (NTA) showed that the *obg* knockdown strain secreted fewer EVs particles and less total EVs protein than the empty‐vector control (EF‐vec), although EVs size distribution was unchanged (Figure S5B,C). Furthermore, we compared the protein composition of EVs by WB and mass spectrometry. The Coomassie Blue staining showed there was no significant difference in terms of the overall protein pattern between EF‐EVs and EF‐dCas‐Obg‐EVs samples except for the obvious difference in Obg protein content (Figure S5D). We then further analysed the protein profiles by mass spectrometry. The results showed that the key proteins were commonly shared. Several unique and differential expressed proteins were identified, including Obg. Subsequent functional enrichment prediction demonstrated that EF‐dCas‐Obg‐EVs exhibited reduced biosynthesis and degradation of amino acids, RNA and other biomolecular processes (Figure S5E‐H). We compared the GTPase activity of EVs using a Colorimetric GTPase Activity Assay Kit (P2435S, Beyotime Biotechnology, Shanghai, China). The results showed that the GTPase activity of EF‐EVs was significantly higher than that of EF‐dCas‐Obg‐EVs, indicating that Obg knockdown significantly inhibits EF‐EVs GTPase activity of Obg. Additionally, we purified the EF‐Obg protein and directly detected its GTPase activity (Figure S5I‐K). Taken together, EF‐Obg was delivered in an active state.

Further experiments demonstrated that the EF‐dCas9‐Obg strain lost its ability to promote HCC cell proliferation compared to the EF‐vec control strain (Figure 5G). Immunoblotting confirmed that infection, conditioned medium, or purified EVs from the EF‐dCas9‐Obg strain no longer activated the mTOR pathway, in contrast to their counterparts from the EF‐vec strain (Figure 5H). Consistently, EVs isolated from the EF‐dCas9‐Obg strain showed compromised ability to promote liver tumour growth in mice, whereas EVs from the EF‐vec strain retained this activity (Figure 5I). IHC staining of tumours further confirmed that EF‐dCas9‐Obg EVs did not induce mTOR pathway activation (Figure 5J). 16S rRNA sequencing of faecal samples revealed that colonisation with the EF‐dCas9‐Obg strain altered gut microbiota composition relative to the EF‐vec strain (Figure S6A–C). Notably, it increased the relative abundance of the probiotic genus *Akkermansia* (Figure S6D,E), indicating that *obg* knockdown remodels the host microbial environment. Together, these findings establish a causal role for *E. faecalis* in hepatocarcinogenesis and demonstrate that knockdown of the *obg* gene attenuates its ability to activate mTOR and promote liver cancer progression.

### 3.7 Elevated EF‐Obg Expression in Liver Tumour Tissues Is Associated With a Poor Prognosis in HCC Patients

**FIGURE 6:**
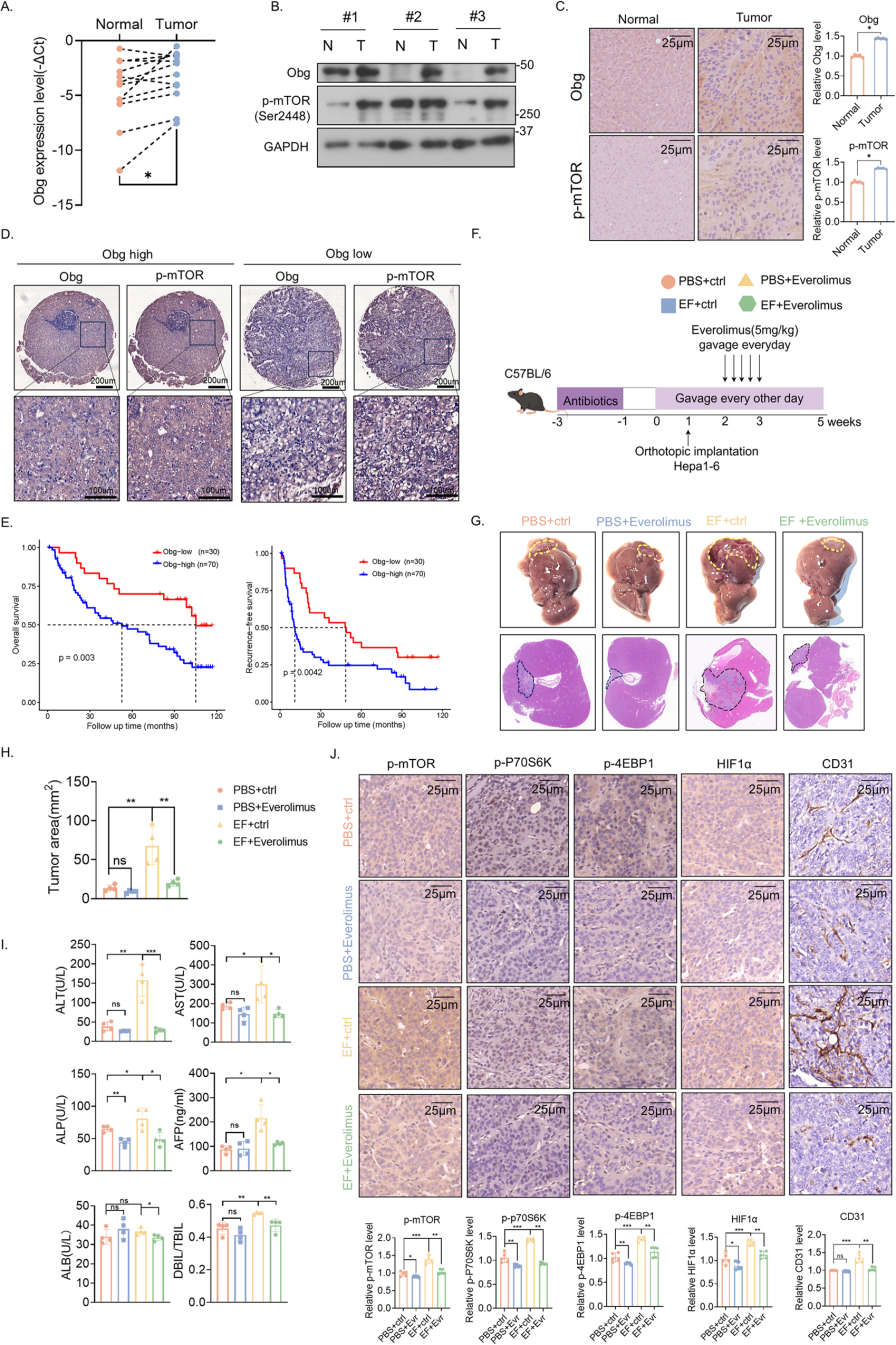
High EF‐Obg expression in liver cancer correlates with poor survival and is therapeutically targetable by the mTOR inhibitor Everolimus. (A) Relative expression of EF‐Obg in tissues based on qPCR. (B) Protein levels of EF‐Obg and p‐mTOR in paired liver tumour tissues and normal tissues based on immunoblotting. N, normal tissues; T, liver tumour tissues. (C) Representative images of immunohistochemical staining of EF‐Obg (using anti‐EF‐Obg antibody) and p‐mTOR in tissues. Scale bar = 25 µm. (D) Representative IHC staining images showing high and low expression of EF‐Obg in human HCC and normal tissues from tissue microarray (TMA). The matched p‐mTOR staining was shown on the right. (E) Kaplan‒Meier survival curves were generated to estimate the overall survival and recurrence‐free survival differences between Obg‐high and Obg‐low groups using a log‐rank test.  *n* = 100. (F) Everolimus treatment scheme of EF‐mediated liver tumourigenesis. Everolimus or control was orally administered to the mice every day, 1 week after the tumour implantation. (G) Representative images of the Everolimus‐treated tumours under *E. faecalis* administration, H&E staining of the tumours (*n* = 4). (H) Quantification of tumour area. (I) Serum levels of ALT, AST, AFP, ALP, ALB and the rate of DBIL/TBIL after indicated treatments. (J) Representative images of immunohistochemical staining from tumour samples of indicated group. Quantification of IHC staining was shown as bar graphs (right). Scale bar = 25 µm. Paired *t*‐test was used in (A). Unpaired two‐tailed Student's *t* test was used in (C). Log rank test was used to calculate the *p* value in (E). One‐way ANOVA test was used in (H), (I) and (J).

To evaluate the clinical relevance of our findings, we examined EF‐Obg expression in liver tumour tissues and matched adjacent normal tissues. Quantitative PCR (qPCR) analysis demonstrated that *obg* gene expression was significantly elevated in HCC (Figure 6A). We generated a monoclonal antibody targeting the EF‐Obg peptide GMVAFRREKYVPD using hybridoma technology (Figure S7A–D) and validated its sensitivity and specificity on tissue sections from prior animal experiments (Figures 2H, 3I; Figure S7E). Additionally, we performed western blot analysis to verify the specificity of the Obg‐specific polyclonal antibody in *E. faecalis* lysates and EF‐EVs. A specific band at approximately 47 kDa was detected in samples. Pre‐incubation of the antibody with its immunogenic peptide abolished this band, confirming specific blocking and binding. Ponceau S staining was performed as input control. These results validate the specificity of the Obg antibody for subsequent applications including immunohistochemistry (Figure S7F). Immunoblotting and immunohistochemistry (IHC) revealed higher EF‐Obg levels in tumour tissues than in normal tissues, which corresponded with elevated phospho‐mTOR (p‐mTOR) expression (Figure 6B,C). Using this antibody, we assessed EF‐Obg expression in a cohort of 100 paired HCC and normal tissue samples. Patients with high EF‐Obg expression exhibited high p‐mTOR levels and significantly shorter overall survival and recurrence‐free survival compared to those with low expression (Figure 6D,E). Furthermore, EF‐Obg levels correlated positively with HCC clinicopathological features, including tumour number, size, TNM (Tumour, Node, Metastasis) stage and BCLC (Barcelona Clinic Liver Cancer) stage (Table S1).

### 3.8 The EF‐Mediated Activation of mTOR in Liver Cancer Can be Therapeutically Targeted Using the mTOR Inhibitor Everolimus

Given that Everolimus, a rapalog, is a potent mTOR kinase inhibitor, we investigated its therapeutic efficacy against liver cancer in the presence of *E. faecalis* colonisation. We established an orthotopic liver cancer model (Hepa1‐6) in C57BL/6 mice, which were then orally gavaged with *E. faecalis*. Mice were subsequently treated with Everolimus to evaluate its effect on tumour progression (Figure 6F). In mice colonized with *E. faecalis*, Everolimus significantly suppressed tumour growth and attenuated liver damage, as indicated by reduced tumour burden and lower serum levels of alanine aminotransferase (ALT) and aspartate aminotransferase (AST). In contrast, Everolimus showed no significant therapeutic effect in mice without *E. faecalis* colonization (Figure 6G–I). Immunohistochemistry confirmed that Everolimus effectively inhibited mTOR pathway activation in the *E. faecalis*‐colonised model (Figure 6J). These data identify EF‐Obg as a potential predictive biomarker and suggest that Everolimus, by suppressing the EF‐Obg‐mTOR signalling axis, may represent a targeted therapeutic strategy for liver cancer patients with *E. faecalis* colonization.

## 4 Discussion

Although growing evidence links gut microbiota to hepatocellular carcinoma (HCC) via the gut‐liver axis (Yu and Schwabe 2017; Ma et al. 2018), the direct impact and underlying mechanisms remain largely unknown. In this study, we demonstrate that tumour‐resident *E. faecalis* activates mTOR signalling by secreting extracellular vesicles containing the bacterial GTPase, Obg. Our findings provide new insight into how *E. faecalis* communicates with host cells and reveal a microbiota‐dependent mechanism of mTOR activation that promotes HCC tumourigenesis—highlighting a previously unrecognised microbial regulator of this critical oncogenic pathway.

Although *E. faecalis* has been identified as highly enriched in the feces of patients with liver diseases, its colonization is not limited to the gut; it is also found within various cancer tissues (Fu et al. 2022; Nunez et al. 2022). However, the specific mechanisms governing its interaction with host cells, particularly tumour cells, remain poorly understood. Our findings confirm that *E. faecalis* is abundant in HCC tissues—a clinically significant observation that suggests its potential as a diagnostic and prognostic biomarker (Figure 1). In our study, subcutaneous xenograft and orthotopic liver tumour models were established, *E. faecalis* was detectable by FISH in the *E. faecalis*‐administered group, further verifying its ability to colonise and translocate (Figure S8A). While other uncharacterised bacterial species in our study may also promote HCC and serve as tumour markers, their roles require further exploration.

We demonstrate that EF‐EVs exert oncogenic effects in liver cancer development, specifically by regulating the host mTOR pathway. It is difficult to definitively determine the exact source of *E. faecalis* that secretes EF‐EVs, we believe that *E. faecalis* from both the intestine and HCC tissues jointly exert their effects in the progression of HCC. Our study is based on the finding that *E. faecalis* is enriched in HCC tissues; meanwhile, existing literatures have reported that more abundant *E. faecalis* colonisation is also detected in the faeces of HCC patients (Iida et al. 2021). Further investigation into other pathways potentially modulated by *E. faecalis*—such as those involving RelA and STK33, as indicated in our study (Figure 2)—could be critical for understanding HCC tumourigenesis. Determining these mechanisms will be essential, as interventions targeting intratumoural microbiota regulation hold considerable promise for rational cancer therapy.

Extracellular vesicles (EVs) serve as essential mediators of host–bacterial communication by delivering proteins and nucleosides (Yu et al. 2017; Wang et al. 2020; Jiang et al. 2023; Choi et al. 2017). Here, we demonstrate that EF‐EVs function as key biological shuttles in interkingdom signalling, transferring bacterial molecules to host cells. In particular, our study reveals that EF‐EVs deliver cargo implicated in mTOR signalling, most notably the bacterial GTPase EF‐Obg, which we show is delivered in a GTP‐bound state and can directly regulate mTOR. Structurally, EF‐Obg contains conserved sequence motifs within its Ras‐like G domain (G1), a region involved in guanine nucleotide binding and hydrolysis. Obg homologs of other bacteria which exhibits different G1 domain failed to activate mTOR signalling pathway. In bacteria such as *Caulobacter crescentus*, guanine nucleotide binding is essential for viability, and Obg homologs are known to regulate bacterial persistence under nutrient starvation (Verstraeten et al. 2015). This functional parallel is intriguing, given mTOR's central role in nutrient sensing and cellular metabolism. Importantly, our modelling indicates that EF‐Obg can bind mTOR in a manner analogous to the host GTPase Rheb. Unlike typical eukaryotic GTPases, however, Obg proteins exhibit exceptionally high guanine nucleotide exchange rates—estimated to be 10^3^–10^5^ times faster than those of Ras‐like GTPases, and potentially faster than Rheb itself (Verstraeten et al. 2011). This raises the possibility that EF‐Obg could activate mTOR more rapidly and potently than host‐derived Rheb, thereby driving efficient mTOR activation and accelerating hepatocellular carcinoma growth.

In a mouse liver cancer model, extracellular vesicles (EVs) derived from *E. faecalis* with *obg* knockdown reduced mTOR activity compared to those from wild‐type bacteria. This indicates that EF‐Obg's positive regulation of mTOR can be replicated in vivo. Notably, a structural human homolog exists: human Obg‐like ATPase 1 (hOLA1), which is overexpressed in various cancers—including colorectal, gastric, lung, ovarian and uterine malignancies (Koller‐Eichhorn et al. 2007; Sun et al. 2010). hOLA1 is known to regulate protein synthesis, modulate the integrated stress response (ISR), and act as a GSK3β inhibitor (Xu et al. 2016). However, whether hOLA1 influences mTOR signalling, or whether EF‐Obg interacts with hOLA1 to contribute to other oncogenic processes, remains to be determined.

Hepatocellular carcinoma (HCC) develops from the progressive accumulation of genomic and epigenomic alterations, which dysregulate key signalling pathways such as AKT/mTOR (Rebouissou and Nault 2020). Activation of the PTEN/PI3K/Akt/mTOR axis and its downstream target ATP‐binding cassette subfamily C member 4 (Abcc4) is known to contribute to hepatocarcinogenesis (Luo et al. 2021). However, the mechanisms driving mTOR activation in HCC remain incompletely understood, particularly in the context of the tumour microbiota. In this study, we generated an EF‐Obg‐specific antibody and used it to detect Obg expression in liver tumour tissues. Our results demonstrate that EF‐Obg expression levels correlate with both mTOR activation and poor overall survival in HCC patients. These findings suggest that EF‐Obg expression and mTOR activity may serve as parallel prognostic biomarkers and that our antibody could be developed into a diagnostic tool.

Given that mTOR inhibition suppresses HCC growth and improves survival (Semela et al. 2007), mTOR inhibitors represent a promising therapeutic strategy. In this study, we specifically explored the effect of the mTOR kinase inhibitor everolimus in liver cancer models colonised by *E. faecalis*. Because the EF‐Obg axis activates mTOR to drive cancer protein translation and angiogenesis, we hypothesised that everolimus would show heightened efficacy in EF‐colonised tumours. Consistent with this premise, everolimus effectively inhibited *E. faecalis*‐induced mTOR activity in liver cancer. These findings highlight the therapeutic relevance of targeting mTOR in HCC patients with tumour‐resident *E. faecalis*. Furthermore, screening for inhibitors that disrupt the EF‐Obg–mTOR interaction—such as competitive peptides—could offer an additional targeted approach for treating EF‐colonised HCC.

In conclusion, our findings reveal an unconventional mechanism of mTOR activation in hepatocellular carcinoma (HCC), mediated by a bacterial GTPase. Specifically, we demonstrate that the *Enterococcus faecalis*–derived protein EF‐Obg directly activates mTOR signalling, thereby illustrating a novel paradigm of cross‐kingdom regulation in tumour progression. This work establishes EF‐Obg as a critical microbial driver of mTOR dysregulation during HCC development. Consequently, therapeutic strategies that inhibit this pathway—such as treatment with everolimus—represent a rational and promising approach for HCC characterised by tumour‐resident *E. faecalis*.

## Author Contributions

Mong‐Hong Lee and Xiangqi Meng conceived and designed the research. Ning Ma, Xiaoshan Xie and Jiarui Wang performed most of the biochemical and molecular experiments, with the assistance of Xiaoling Huang, Yue Wei, Qihao Pan, Boyu Zhang, Jiaying Zheng and Peng Zhang. Zhikai Zheng and Zhongguo Zhou ascertained and processed clinical specimens. Zhikai Zheng performed the bioinformatics analysis. Ning Ma and Haidan Luo constructed the Obg knockdown strain by CRISPR interference under Xue Liu's guidance. Zhi‐Min Zhang predicted Obg‐mTOR binding/interactions domain. Ning Ma, Jiarui Wang, Xiaoshan Xie, Huilin Jin and Xijie Chen performed mice experiments. Ning Ma and Jiarui Wang conducted the bioinformatics analyses. Xijie Chen, Xiaoling Huang, Qihao Pan, Yue Wei, Boyu Zhang, Jiaying Zheng and Fenghai Yu contributed to discussion and data interpretation. Ning Ma, Xiangqi Meng and Mong‐Hong Lee wrote the manuscript. All authors have read and approved the final manuscript.

## Funding

This work was supported by the National Natural Science Foundation of China (82373139, 82273133 and 82303028), Guangdong Basic and Applied Basic Research Foundation(2023A1515030261 and 2025A1515012612), Guangzhou Municipal Science and Technology Program Key Project (202206010167) and the National Key Research and Development Program of China (2020YFA0803300).

## Ethics Statement

Forty‐one fresh‐frozen paired samples of liver cancer and para‐cancerous tissue, as well as paraffin‐embedded liver cancer tissue microarrays (TMA), were collected from the Department of Surgery at Sun Yat‐sen University Cancer Center. All samples were collected with the patients’ written informed consent and with approval from the Institutional Review Board of the Sixth Affiliated Hospital of Sun Yat‐sen University (ID: 2024ZSLYEC‐212). All mice were purchased from the Gempharmatech (Guangdong) and were maintained under standard laboratory conditions, with free access to food and water. The animal experiments were conducted in accordance with protocols approved by the Institutional Animal Care and Use Committee of Sun Yat‐sen University (No. D2022‐0155XS) and the Institutional Animal Care and Use Committee of South China Agricultural University (No. 2023C090). The detailed design of animal experiments can be found in supplemental methods.

## Consent

All samples were collected with the patients’ written informed consent.

## Conflicts of Interest

The authors declare no conflicts of interest.

## Supporting information




**Supporting Information**: jev270323‐sup‐0001‐SuppMat.docx

**Supporting Information**: jev270323‐sup‐0002‐SuppMat.docx

## Data Availability

Protocol details, further information and requests for resources and reagents can be directed by the lead contact Prof Mong‐Hong Lee (limh33@mail.sysu.edu.cn). The 16S data, transcriptome data and mass spectrometry proteomics data will be further uploaded. This paper does not report original code.
