# Supplementary material for: Enterococcus faecalis Extracellular Vesicles Deliver the Bacterial GTPase Obg to Hijack mTOR Signalling in Hepatocellular Carcinoma
Source: J Extracell Vesicles. 2026 Jun 17;15(6):e70323. doi: 10.1002/jev2.70323 (PMC13275991; doi:10.1002/jev2.70323)

**
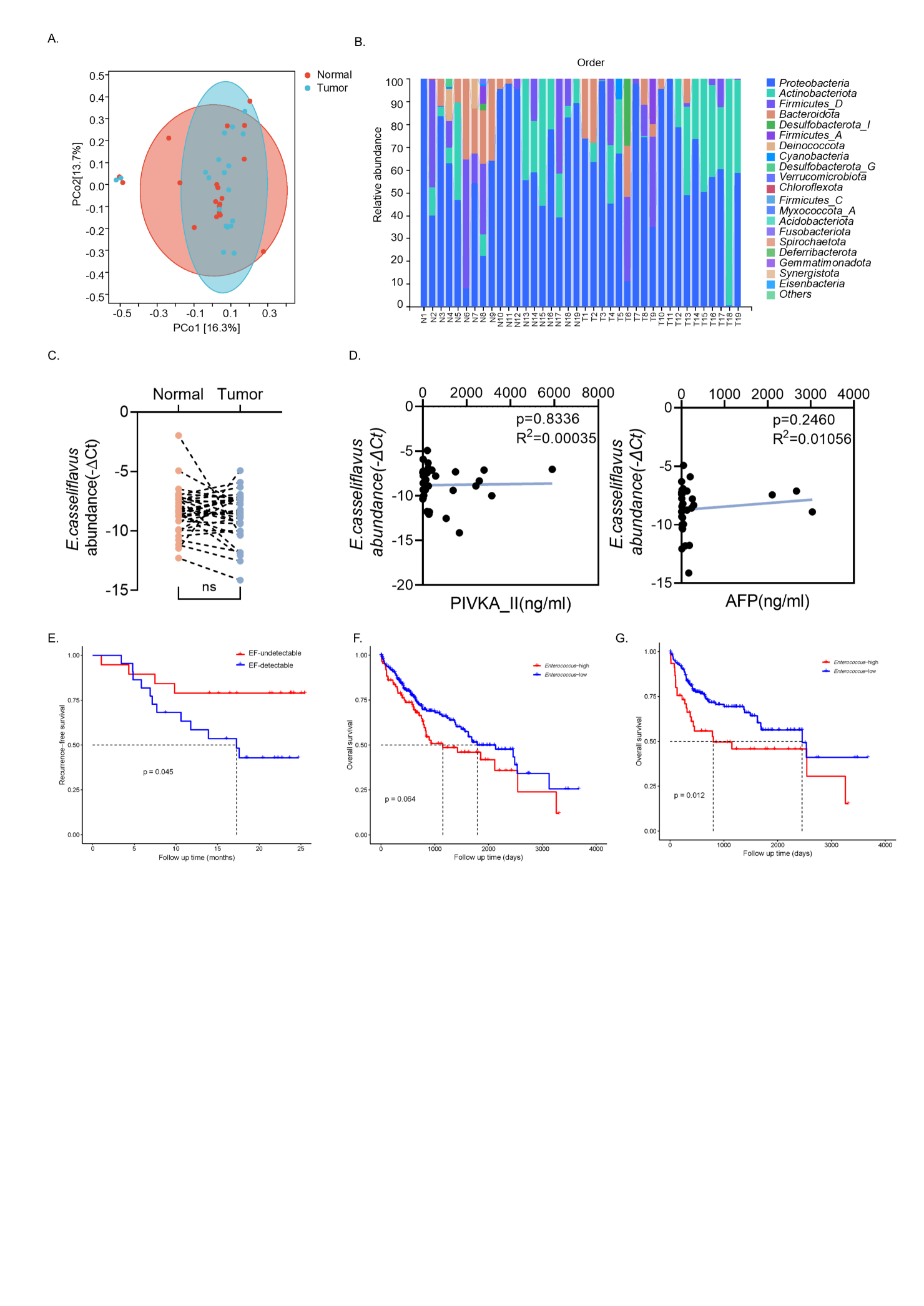
**

**Supplementary Figure 1. The microbial composition of tissue-resident bacteria in liver tumor and adjacent normal tissue.**

(A) Principal Coordinate Analysis (PCoA) based on unweighted UniFrac analysis of extracted DNA from tissue-resident bacteria in tumor and normal tissues. Each dot represents a single sample.

(B) Relative abundance of tissue-resident microbiota in tumor and normal tissues at Order level based on 16S sequencing.

(C) Relative abundance of *E.casseliflavus* in liver tumor tissues and normal tissues.

(D) Spearman’s correlation between -ΔCt values of *E.casseliflavus* with serum PIVKA_II level and AFP level in HCC patients.

(E) Kaplan‒Meier survival curve was generated to estimate the recurrence-free survival differences between EF-detectable and EF-undetectable groups using a log rank test. N = 41.

(F) Kaplan‒Meier survival curve was generated to estimate the survival probability differences between *Enterococcus-*high and *Enterococcus*-low groups using a log rank test. N = 366.

(G) Kaplan‒Meier survival curve of HBV-positive HCC patients. N = 221.

ns, not significant. Paired *t*-test was used in (C).


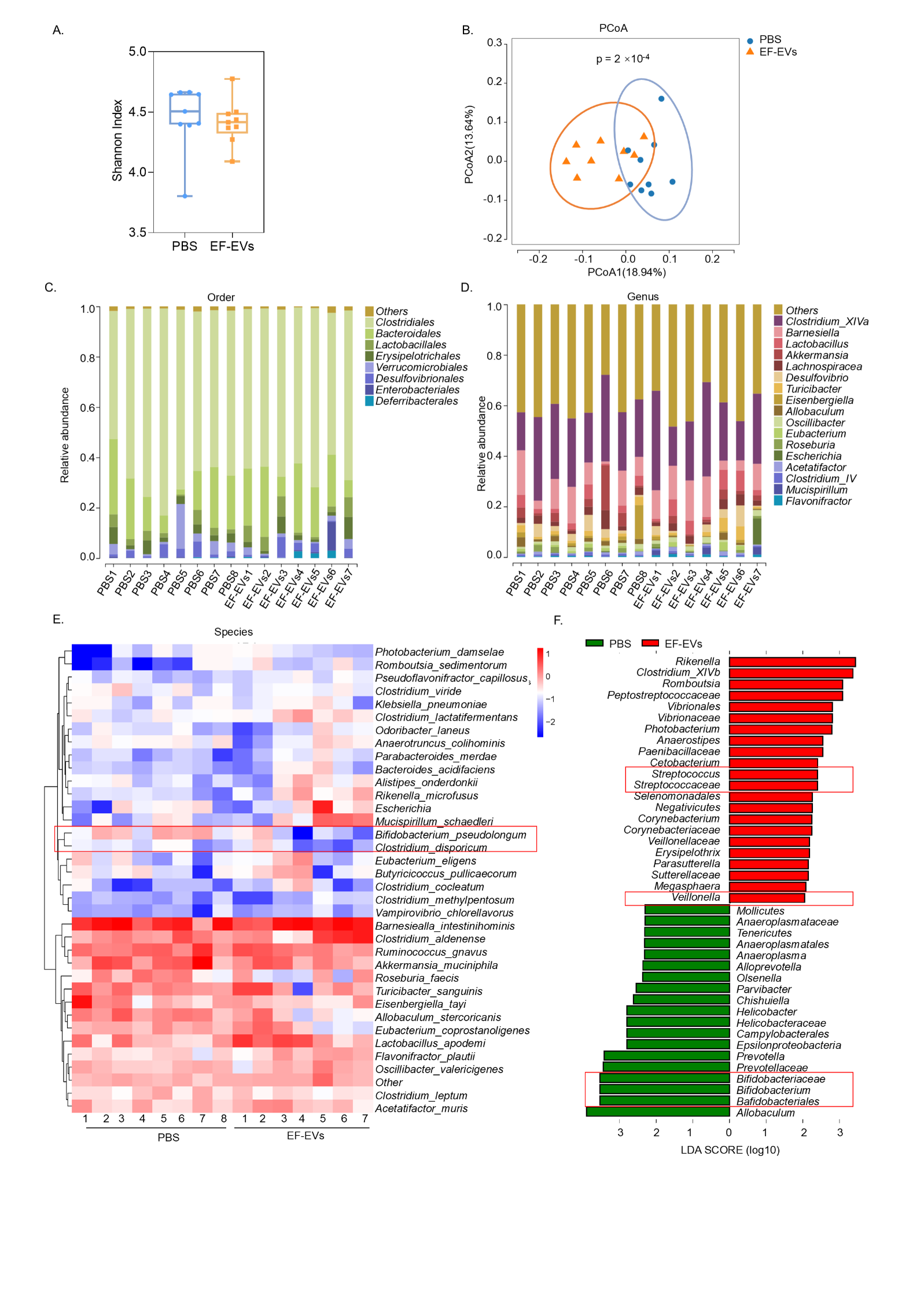


**Supplementary Figure 2. *E. faecalis* EVs alter the gut microbiota composition in mice.**

(A) 16S sequencing of mice feces from EF-EVs or PBS treatment group. Shannon index showed the alpha diversity of microbiota between 2 groups.

(B) PCoA based on un-weighted UniFrac analysis of mice feces microbiota in two groups. Each dot represents a single sample.

(C) Relative abundance of fecal microbiota in samples from EF-EVs or PBS treatment group at Order level.

(D) Relative abundance of fecal microbiota in samples from EF-EVs or PBS treatment group at Genus level.

(E) Heatmap showing fecal bacteria species distribution in samples from EF-EVs or PBS treatment group. The red box highlights species that were significantly decreased in the EF-EVs group.

(F) Linear discriminant analysis (LDA) statistical method was used to identify the most differentially abundant taxa between two groups (LDA > 2 and p < 0.05). The red box highlights altered species that have been reported to be associated with HCC.

Wilcoxon test was used to calculate the *p* value in (A). Unpaired two-tailed Student’s *t* test was used in (B).


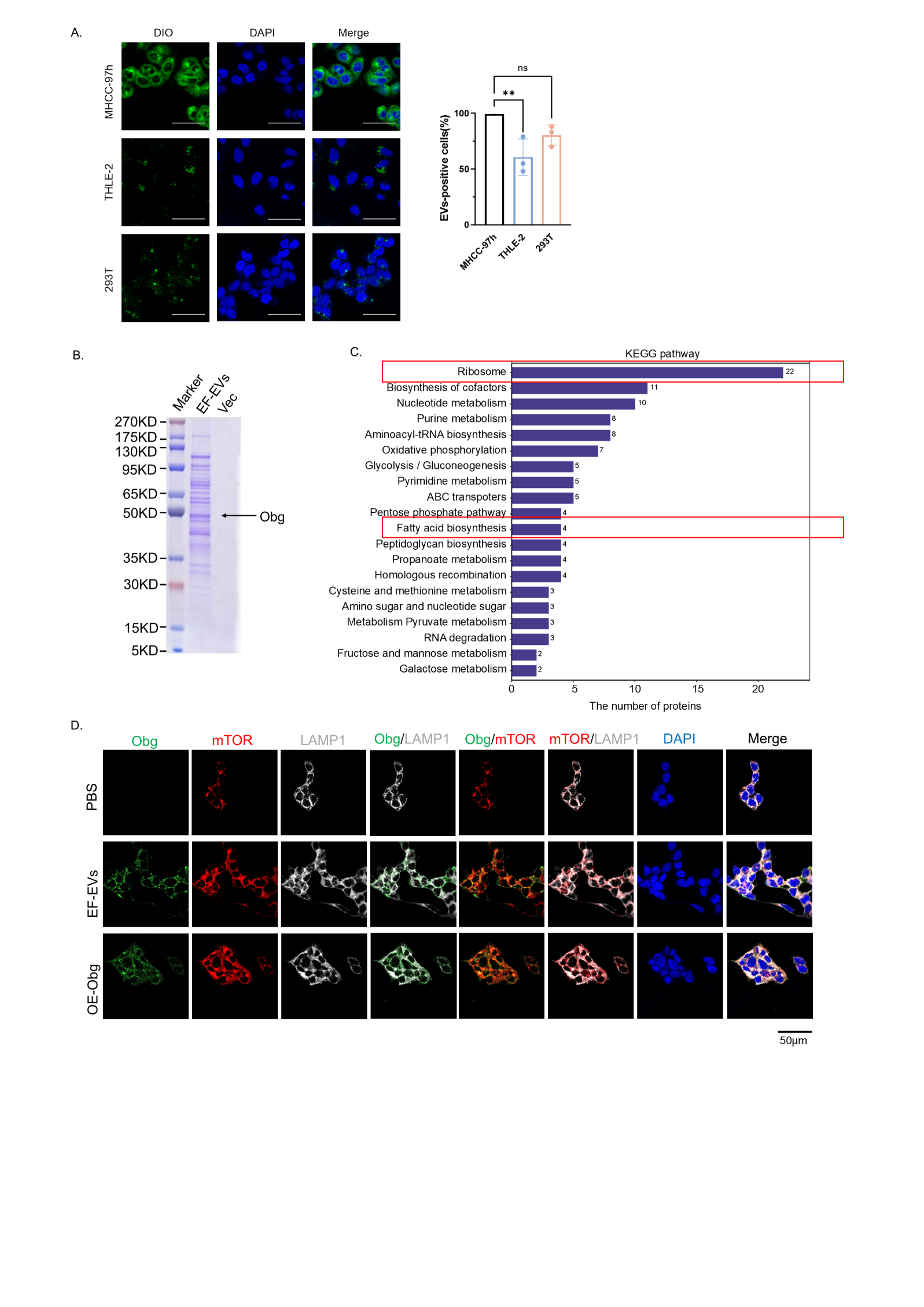


**Supplementary Figure 3. Uptake, proteomic profiling and Obg/mTOR/LAMP1 localization analysis of EF-EVs.**

(A) Confocal micrographs of MHCC-97h, 293T and THLE-2 cells incubated with 20μg/mL DiO-labeled EVs for 120 min. Scale bars = 25 μm. Quantification of EVs-positive cells were shown as bar graphs.

(B) Coomassie Blue staining of EF-EVs protein bands based on SDS-PAGE.

(C) The Kyoto Encyclopedia of Genes and Genomes (KEGG) annotation results of the identified proteins from EF-EVs. The red boxes highlight the most abundant proteins belonging to the ribosome group and fatty acid biosynthesis group.

(D) The dual and multiplex immunohistochemistry analysis of the localization of Obg (green), mTOR(red) and LAMP1 (white) in 293T cells after EF-EVs/overexpression *obg* treatments. Nuclei (blue) were stained with DAPI. Scale Bars = 50 μm.

One-way ANOVA was used in (A). **, *p* < 0.01; ns, no significant.


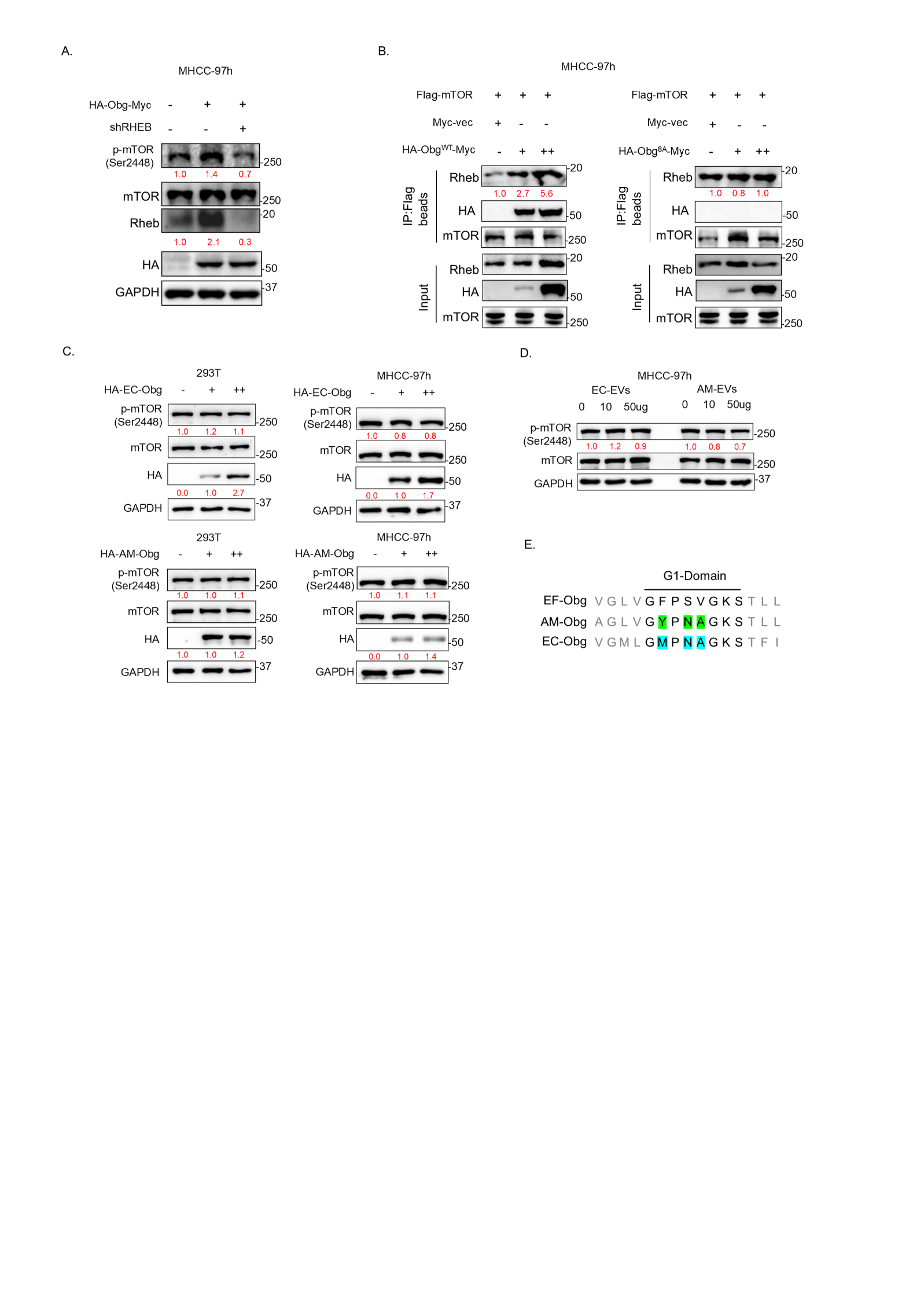


**Supplementary Figure 4. EF-Obg promotes mTOR-Rheb interaction via its G1 domain, while Obg homologs from non-pathogenic bacteria fail to activate mTOR signaling.**

(A) Protein levels of mTOR and its phosphorylation levels in Rheb-knockdown MHCC-97h cells with Obg overexpression. Quantification was shown as red numbers.

(B) The interaction between mTOR and Rheb under dose-dependent overexpression Obg^WT^/Obg^8A^ condition was determined by CO-IP assay. Quantification was shown as red numbers.

(C) Protein levels of mTOR and its phosphorylation levels in 293T and MHCC-97h cells after EC-Obg / AM-Obg overexpression. Quantification was shown as red numbers.

(D) Protein levels of mTOR and its phosphorylation levels in MHCC-97h cells after EC-EVs / AM-EVs treatment. Quantification was shown as red numbers.

(E) Comparison of GTP binding sites 1 (G1) among Obg homologs. Unaligned amino acid sequences in G1 are highlighted.


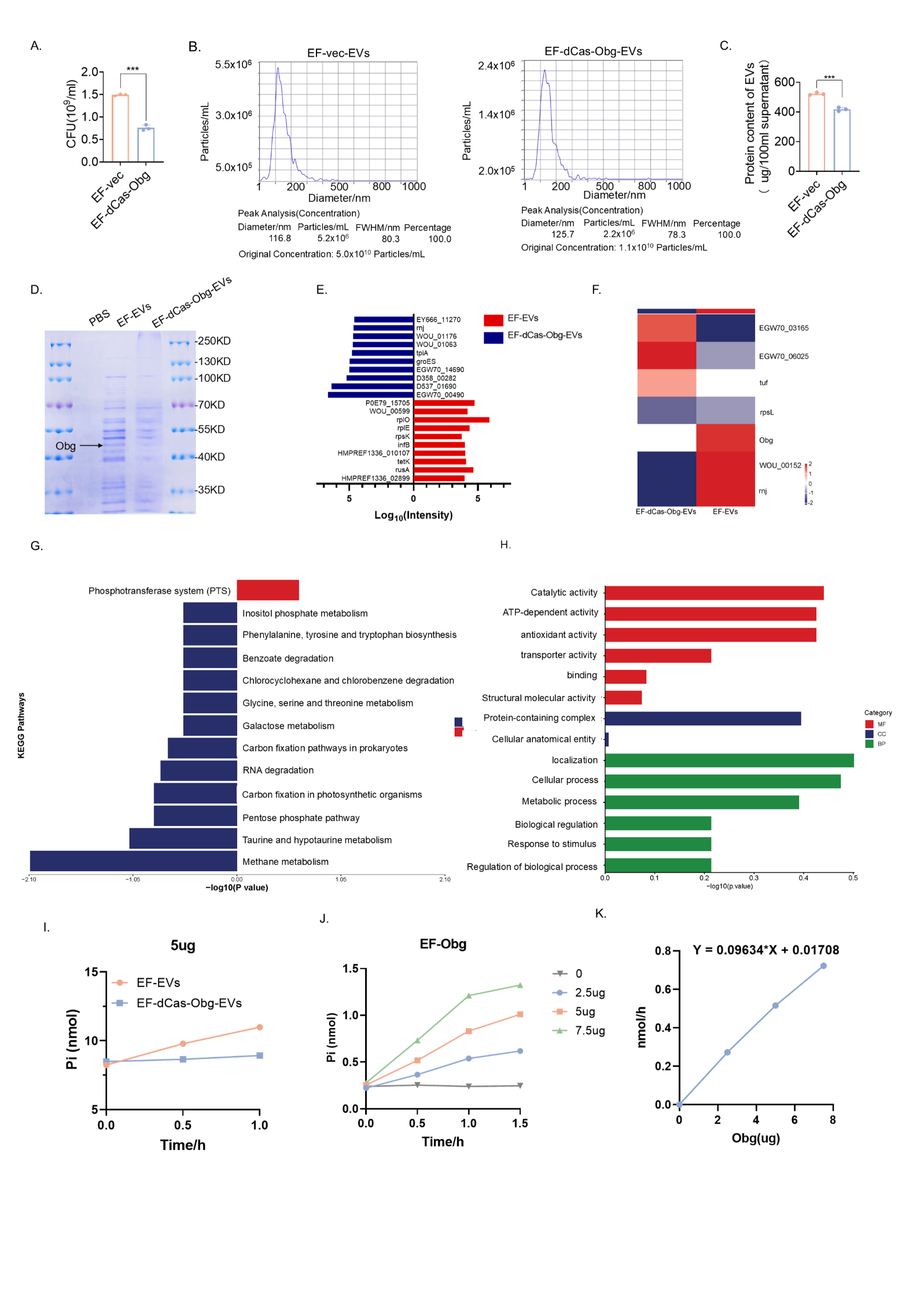


**Supplementary Figure 5. The engineered knockdown strain EF-dCas-Obg exhibits reduced growth rate, extracellular vesicle (EVs) production, and associated biological functions.**

(A) C.F.U calculation of EF-vec and EF-dCas-Obg strains after 12 hours cultured in BHI medium.

(B) Nanoparticle tracking analysis of EVs isolated from EF-vec and EF-dCas-Obg strains after normalizing with the total bacterial content.

(C) The protein concentration of EVs isolated from EF-vec and EF-dCas-Obg strains after normalizing with the total bacteria content.

(D) Coomassie Blue staining of EF-EVs and EF-dCas-Obg-EVs protein bands based on SDS-PAGE.

(E) The top 10 unique proteins with the highest intensity were identified in each group.

(F) Differential expressed proteins between the two groups. *p* < 0.05, log2(FC) > 2.

(G) The Kyoto Encyclopedia of Genes and Genomes (KEGG) annotation results of the differentially proteins between EF-EVs and EF-dCas-Obg-EVs.

(H) The gene ontology (GO) annotation results of the differential proteins between EF-EVs and EF-dCas-Obg-EVs.

(I&J) Schematic diagram showing the molar amount of inorganic phosphate (Pi) released from GTP hydrolysis by different amounts of EVs and Obg protein over time. The slope of the fitted equation (nmol/h) represents the rate of Pi release per hour by samples at the corresponding concentration.

(K) Schematic diagram of the fitted linear curve with the amount of Obg protein(ug) as the X-axis and the molar amount of Pi released per unit time (nmol/h) as the Y-axis. The slope of the fitted line represents the GTPase activity.

Data are presented as mean ± SD; Unpaired two-tailed Student’s *t* test was used in (A), (C). ***, *p* < 0. 001. C.F.U, Colony Forming Units


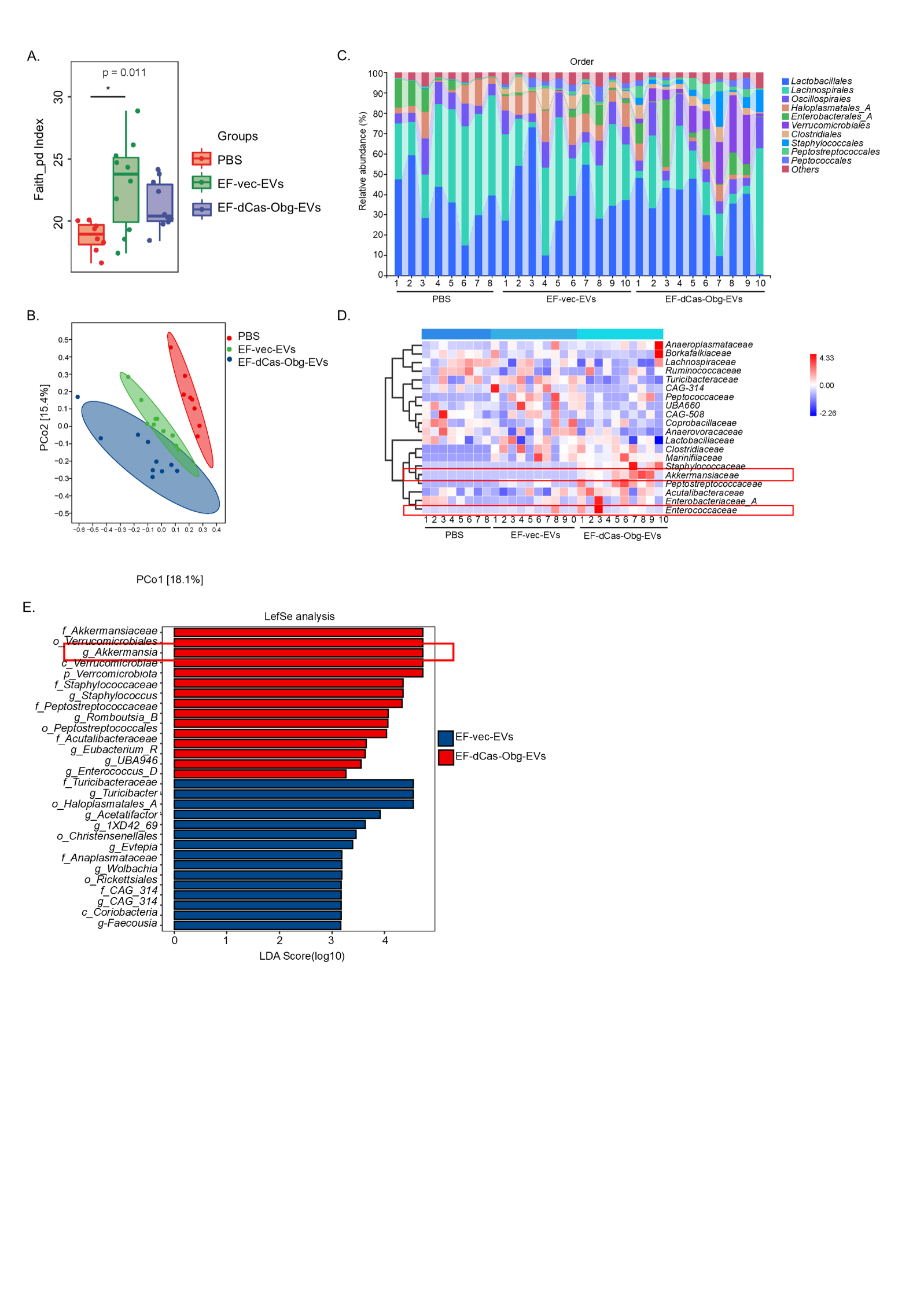


**Supplementary Figure 6. Gut microbiome alterations in mice following colonization with the EF-dCas-obg strain.**

**(**A) Faith_pd index showed the alpha diversity of microbiota in three groups.

(B) PCoA based on weighted UniFrac analysis of bacterial communities in three groups. Each dot represents a single sample.

(C) Relative abundance of mice fecal microbiota in three groups at Order level.

(D) Heatmap showing the distribution of various bacteria families in feces among 3 groups. The red boxes highlight altered bacteria *Akkermansiaceae* and *Enterococcaceae*.

(E) Linear discriminant analysis (LDA) effect size was used to identify the most differentially abundant taxa between two groups (LDA>3). The red boxes highlight increased *Akkermansia* genus.

Wilcoxon test was used to calculate the *p* value in (A). One-way ANOVA test was used in (B). *, *p* < 0.05.

**
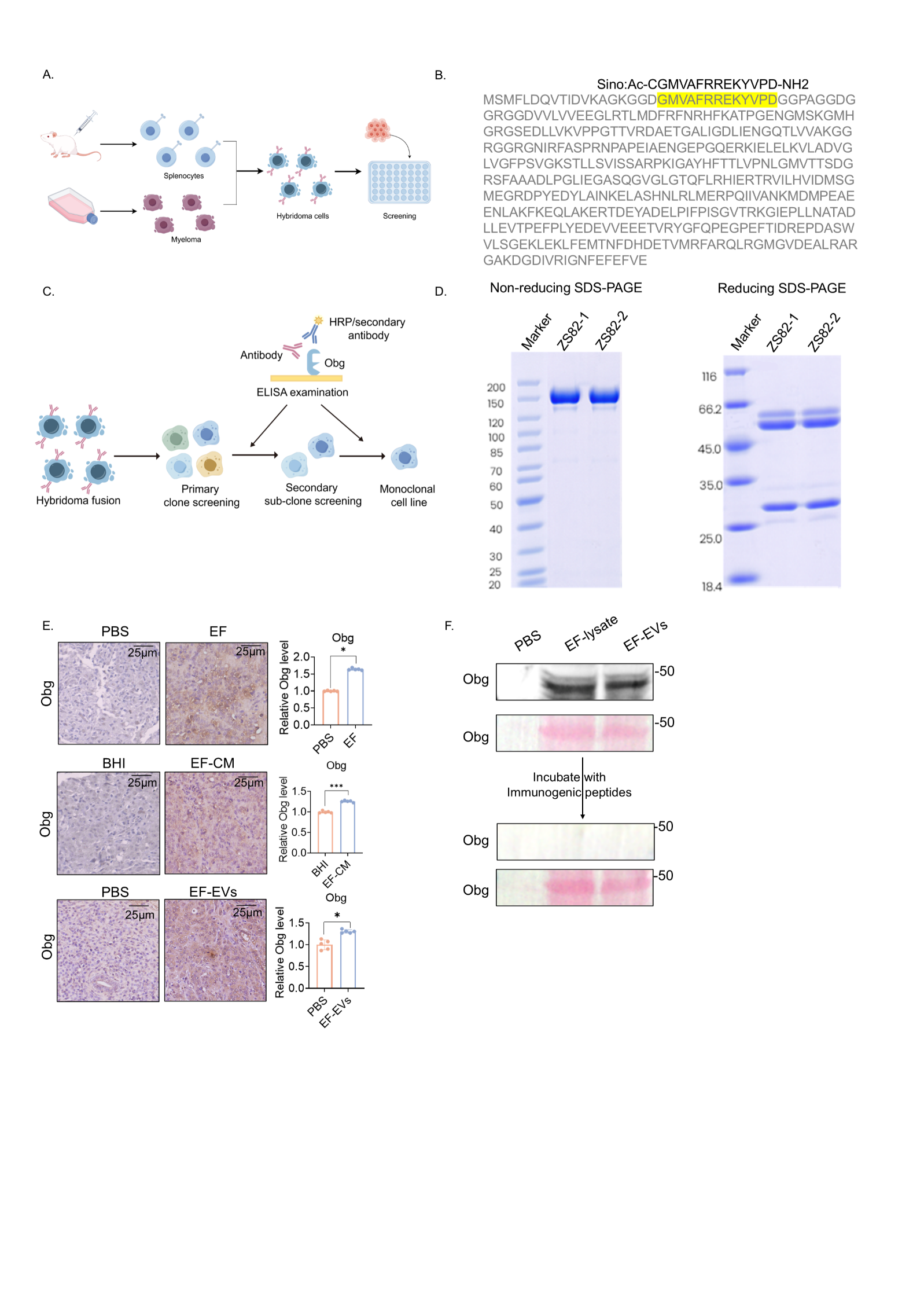
**

**Supplementary Figure 7. Generation of an anti-Obg polyclonal antibody.**

(A) Schematic diagram of anti-Obg antibody production process.

(B) Peptide sequence aligned with Obg immunogen.

(C) Animal immunity and titer detection process.

(D) Non-reducing (left) and reducing (right) SDS-PAGE electrophoresis of purified antibody.

(E) Representative images of immunohistochemical staining of EF-Obg using the prepared anti-Obg antibody. Quantification of IHC staining was shown as bar graphs (below). Scale bar = 25 μm.

(F) Specificity verification of the Obg-specific polyclonal antibody by western blot. Ponceau S staining as input control.

Data are presented as mean ± SD; Unpaired two-tailed Student’s *t* test was used in (E). *, *p* < 0.05; ***, *p* < 0. 001.

**
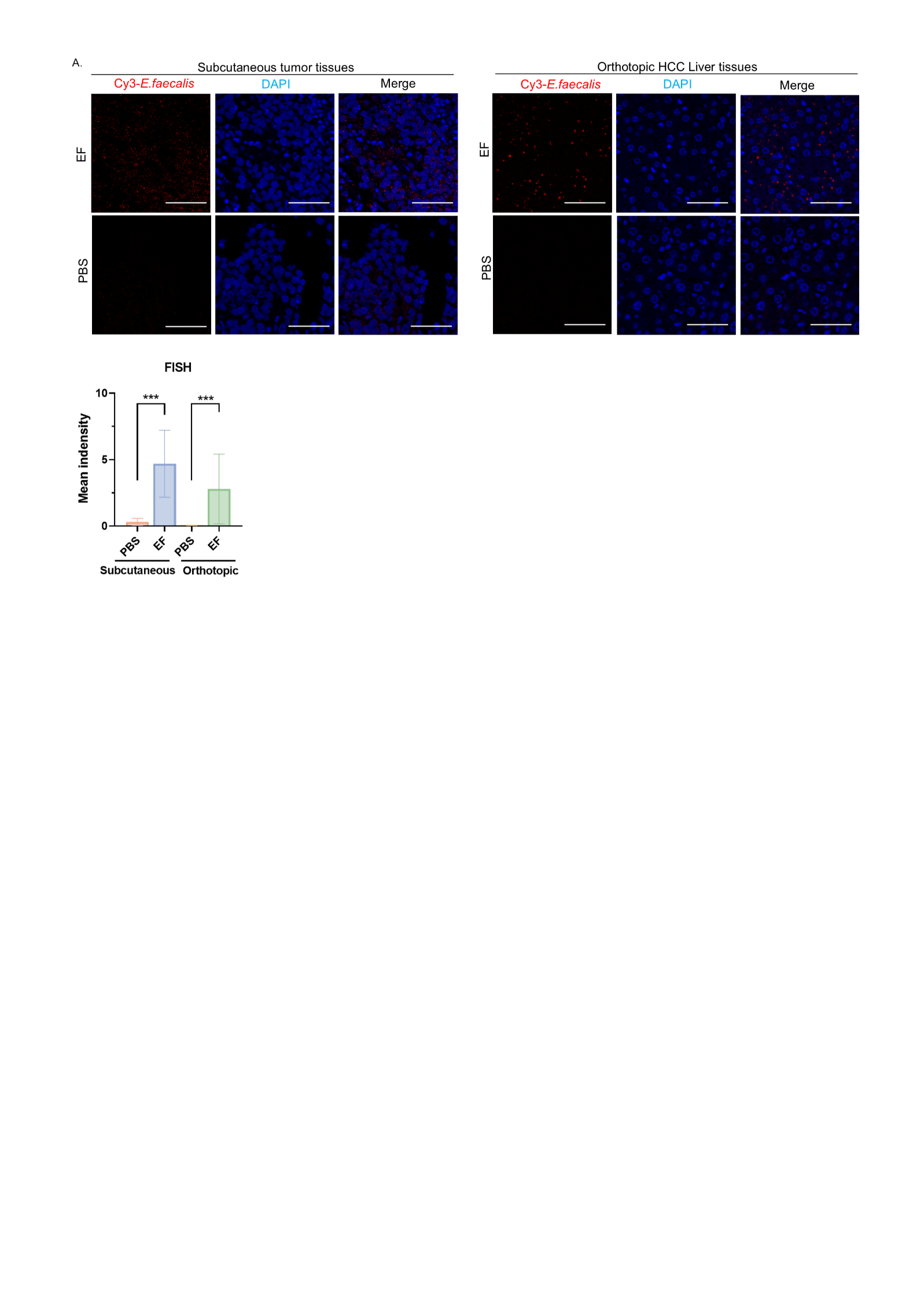
**

**Supplementary Figure 8.** **FISH Detection of *E. faecalis* in tumor-bearing mice tissues.**

(A) Detection of *E. faecalis* in tissues by fluorescence in situ hybridization (FISH). Sections of tissue were counterstained with DAPI (blue) and *E. faecalis* probes (red). Scale bar = 50 μm. Quantification of mean indensity was shown as bar graphs. Unpaired two-tailed Student’s *t* test was used. ***, *p* < 0. 001.


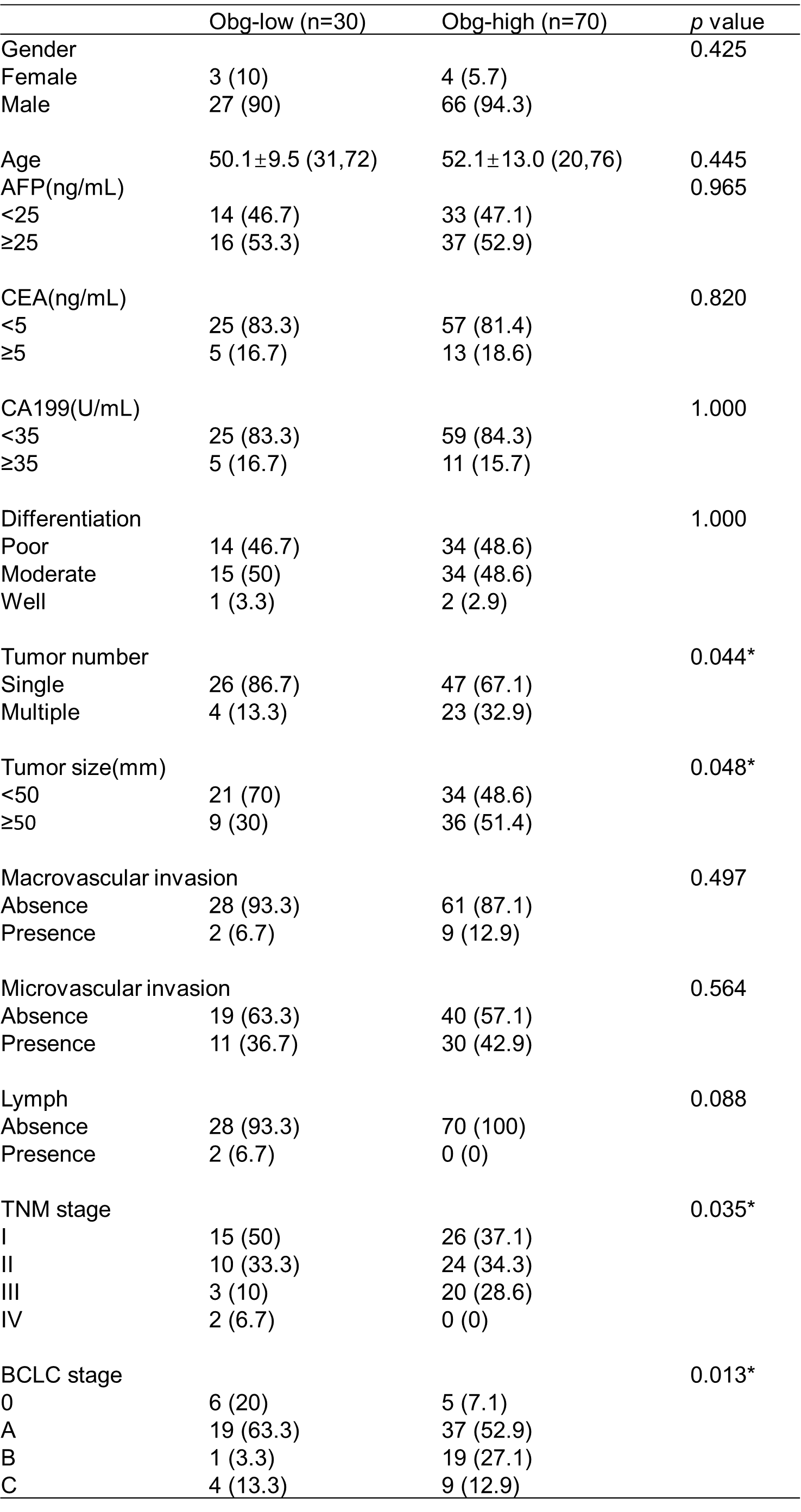
**Supplemental Table 1: Association between EF-Obg expression and clinicopathological features in 100 HCC cases.**

The *p* values were calculated in SPSS19 using a chi-square test. All *p* values were two sided and the level of statistical significance was set at < 0.05. AFP, α-fetoprotein; CEA, Carcinoembryonic antigen; CA199, Carbohydrate Antigen 199.

**Supplemental Table 2: Key reagent and resource**


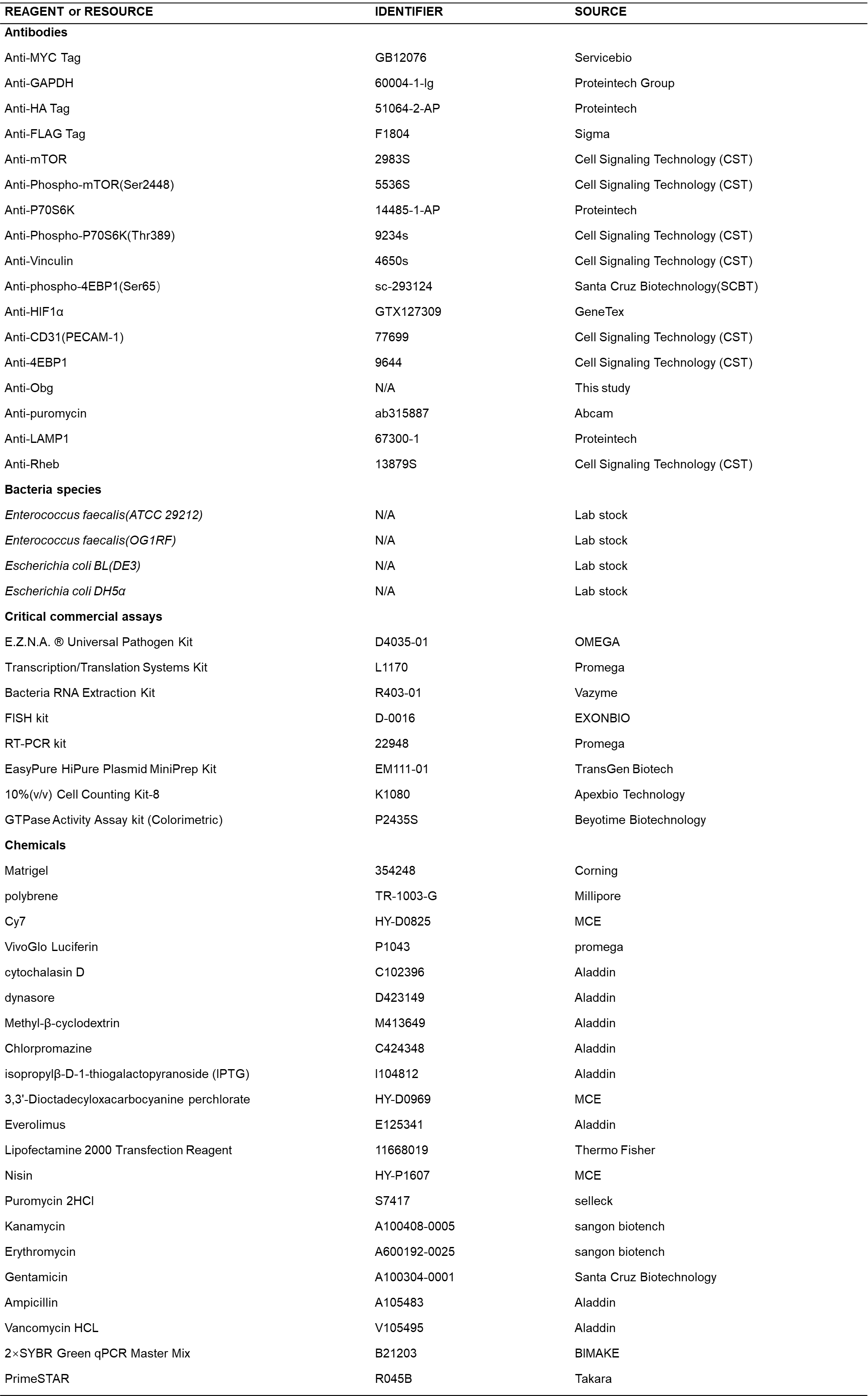


**Supplemental Table 3: Sequences for PCR**


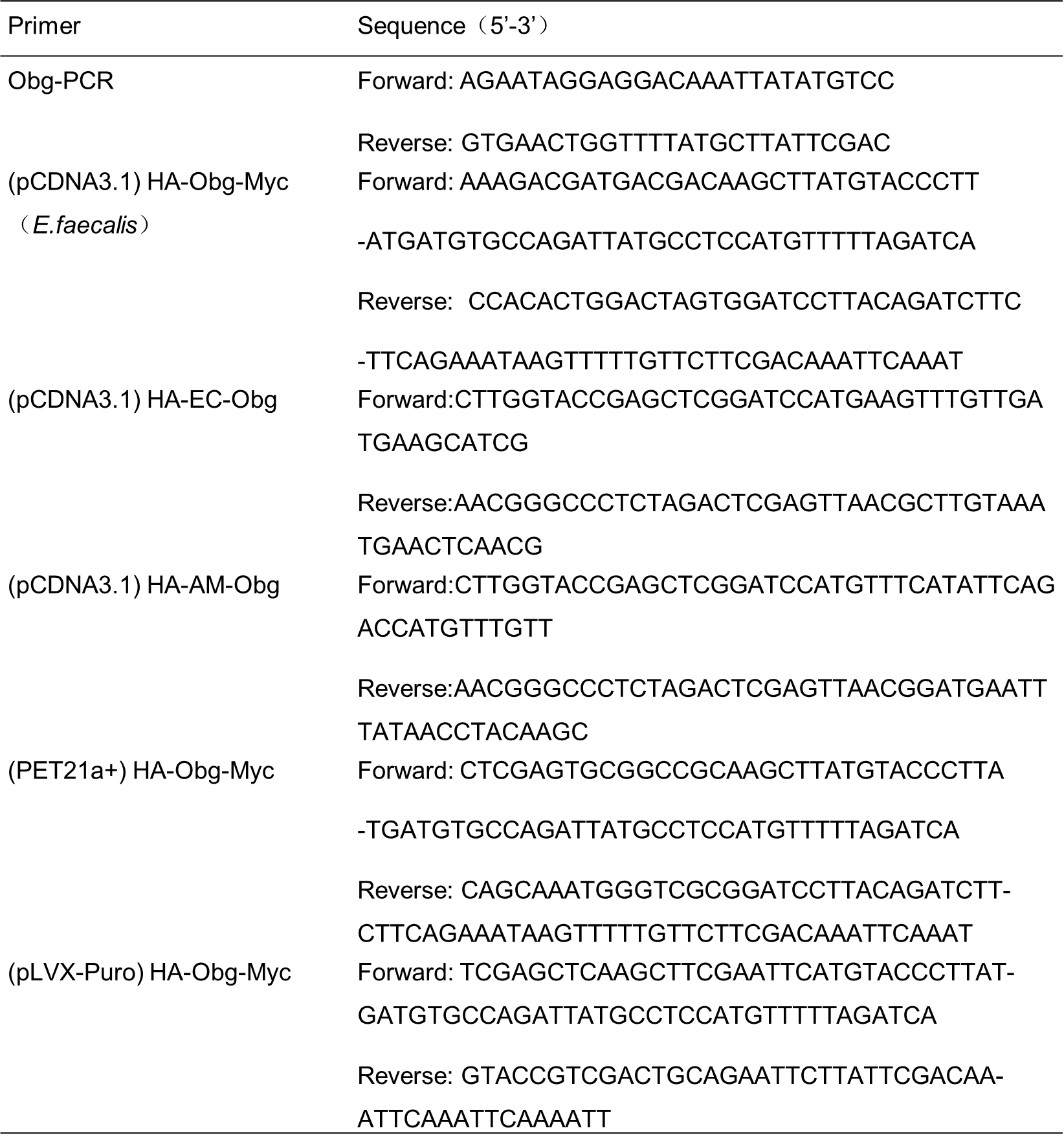


**Supplemental Table 4: Target EF-Obg sequences predicated by CHOPCHOP**


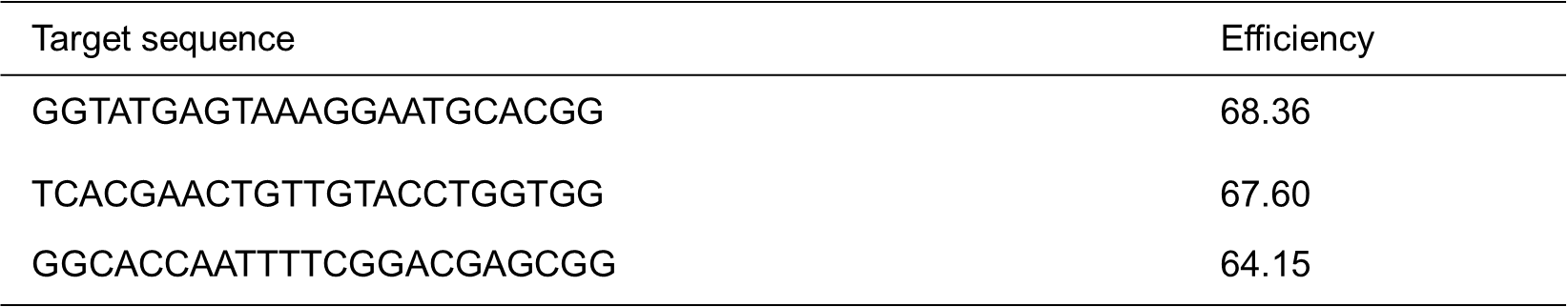


**Supplemental Table 5: Primer sequences for RT-qPCR**


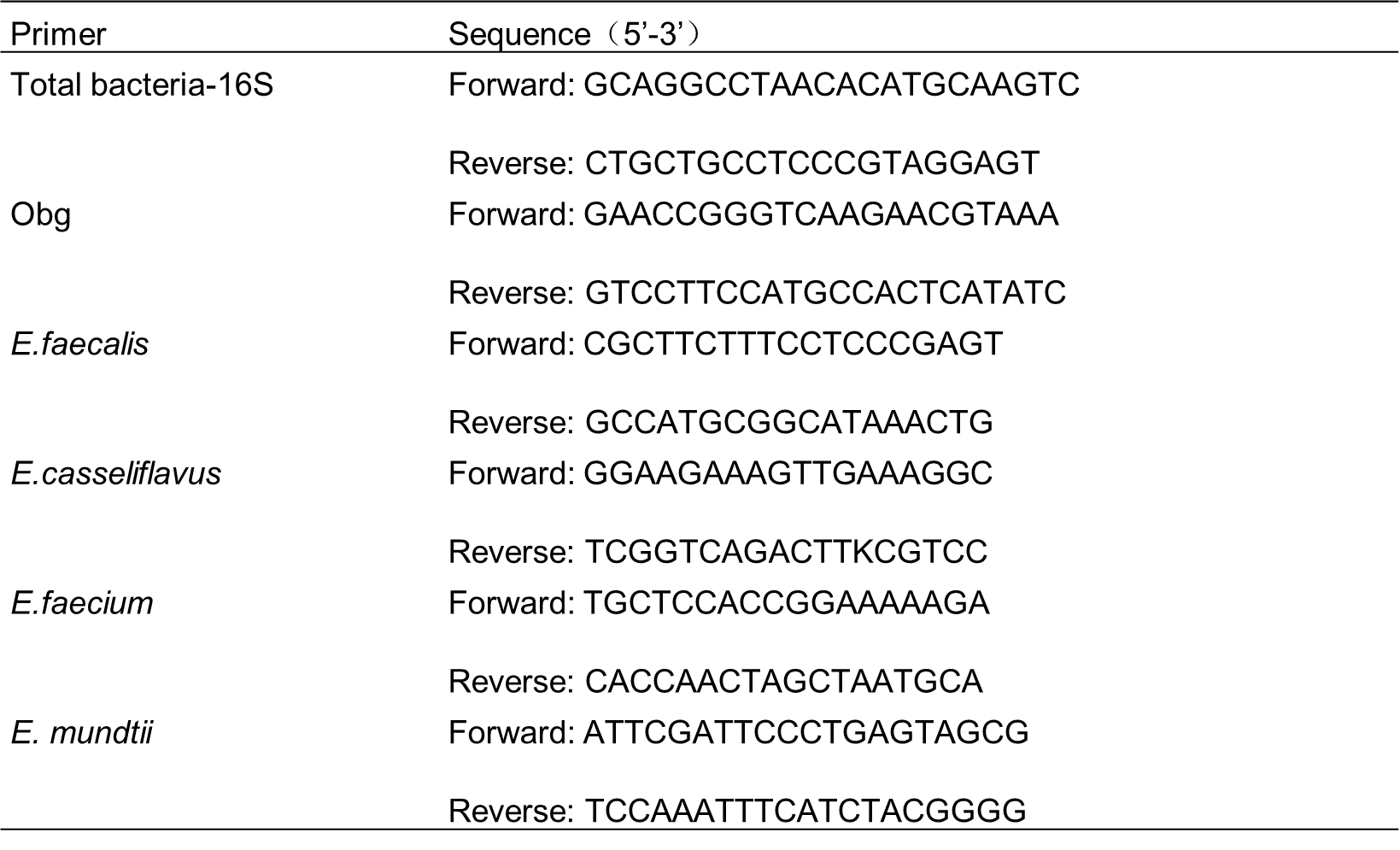


**Supplemental Table 6: Primer sequences for shRHEB**


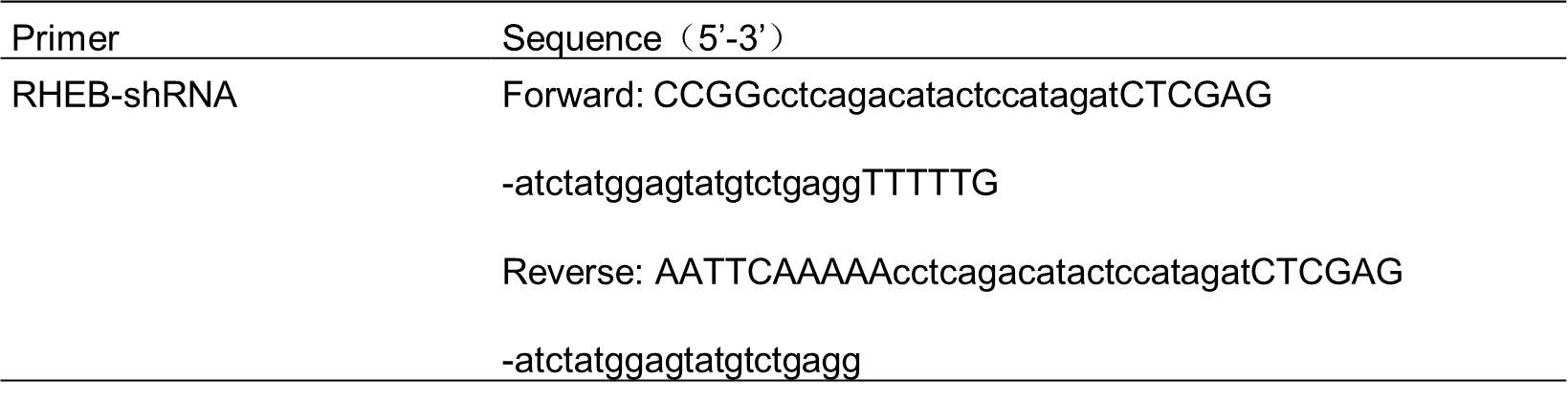

Supplement: Supplementary file 1 — Supporting Information: jev270323‐sup‐0001‐SuppMat.docx [file JEV2-15-e70323-s001.docx]
