# Supplementary material for: Enterococcus faecalis Extracellular Vesicles Deliver the Bacterial GTPase Obg to Hijack mTOR Signalling in Hepatocellular Carcinoma
Source: J Extracell Vesicles. 2026 Jun 17;15(6):e70323. doi: 10.1002/jev2.70323 (PMC13275991; doi:10.1002/jev2.70323)
Supplement: Supplementary file 2 — Supporting Information: jev270323‐sup‐0002‐SuppMat.docx [file JEV2-15-e70323-s002.docx]

**Supplementary methods**

**16S rRNA gene amplicon sequencing**

PCR ampliﬁcation of the bacterial 16S rRNA genes V3–V4 region was performed using the forward primer 338F (5'-ACTCCTACGGGAGGCAGCA-3') and the reverse primer 806R (5'-GGACTACHVGGGTWTCTAAT-3'). Sample-specific 7-bp barcodes were incorporated into the primers for multiplex sequencing. The PCR components contained 5 μl of buffer (5 × ), 0.25 μl of Fast pfu DNA Polymerase (5 U/μl), 2 μl (2.5 mM) of dNTPs, 1 μl (10 μM) of each Forward and Reverse primer, 1 μl of DNA Template, and 14.75 μl of ddH_2_O. Thermal cycling consisted of initial denaturation at 98 °C for 5 min, followed by 25 cycles consisting of denaturation at 98 °C for 30 s, annealing at 53 °C for 30 s, and extension at 72 °C for 45 s, with a final extension of 5 min at 72 °C. PCR amplicons were puriﬁed with Vazyme VAHTSTM DNA Clean Beads (Vazyme, Nanjing, China) and quantiﬁed using the Quant-iT PicoGreen dsDNA Assay Kit (Invitrogen, Carlsbad, CA, USA). After the individual quantification step, amplicons were pooled in equal amounts, and pair-end 2 x 250 bp sequencing was performed using the Illlumina NovaSeq platform with NovaSeq 6000 SP Reagent Kit (500 cycles) at Shanghai Personal Biotechnology Co., Ltd (Shanghai, China).

**Bacteria DNA extraction and qPCR quantification**

E.Z.N.A. ® Universal Pathogen Kit (OMEGA, D4035-01) was used to extract bacteria DNA. Briefly, 35 mg tumor tissue samples were added to a Disruptor Tube with 725 μl SLX-Mlus Buffer and vortexed in a TissueLyser (QIAGEN, 85300) with glass beads to lyse and homogenize the samples. The system combined Omega Bio-tek’s MicroElute® LE DNA Columns with RBB Buffer to eliminate PCR inhibiting compounds within the samples and elute highly concentrated DNA. Total genomic DNA was extracted from the tumor tissue and eluted in 100 μl of Elution Buffer, which was preheated to 70 °C. The primers of *E. faecalis* and total bacteria were listed in the Supplemental Table 5. The abundance of the bacterium was calculated as a relative unit normalized to the total bacteria of that sample (where ΔCt=the Ct value of the target (*E. faecalis*) - the Ct value of total bacteria (16S)), using the 2^-ΔCt^ method ^14^.

**Fluorescence in Situ Hybridization (FISH)**

The *E. faecalis*-specific probe (5’GGTGTTGTAGCATTTCG) labeled with the fluorophore Cy3 was used to detect the bacterial colonization within human tissues by FISH. The hybridization was performed using a FISH Kit (EXONBIO, D-0016). Briefly, paraffin-embedded sections were de-waxed and hydrated first. The slides were washed in PBS for 10 min twice. Then covered the spots with 0.2 M HCl for 15 min at room temperature. Subsequently, the spots were covered with Proteinase K (50 μg/ml) for 30 min at 37°C and washed with PBS. 20 μl of the hybridization buffer containing the specific probe designed for *E. faecalis* was added. Incubated the slides in a dark chamber for 24 h at 46 °C. After hybridization, the sections were rinsed with wash buffer (20 mM Tris-HCl pH7.4, and 0.9 M NaCl) for 3 times (5 min/time). Air dry the slides for 20 min. 20 μl of DAPI (Life Technologies) were covered and incubated for 15 min at room temperature. The images were captured using Leica Laser Scanning confocal microscope (Leica TCS-SP8, Leica Microsystems Inc, Buffalo Grove, IL, USA).

**Detailed animal experiments**

*Subcutaneous mouse model*

SPF C57BL/6 female mice were pre-treated with an antibiotic cocktail (doripenem and vancomycin) for 2 weeks and then randomly divided into different groups as desired. One week later, indicated materials (*E. faecalis* (1×10^8^ CFU resuspended in 200 μl PBS); EF-CM (200 μl); EF-vec-EVs (50 μg in 200 μl PBS); EF-dCas-Obg-EVs (50 μg in 200 μl PBS); PBS (200 μl)) were administered every other day until the end of the experiment. Mice were subcutaneously injected with 1×10^6^ Hepa1-6 cells one week after the start of gavage. To verify the effects of EF-Obg *in vivo*, C57BL/6 female mice were randomly grouped and then subcutaneously injected with 5 × 10^5^ Hepa1-6 cells infected with plvx-Obg viral selection or plvx-vector viral selection. The tumor length and width were measured every 3 days. The volume was calculated according to the formula (length × width^2^)/2.

*Orthotopic mouse model*

To verify the effects of *E. faecalis* EVs *in vivo***,** SPF C57BL/6 female mice were pre-treated with an antibiotic cocktail for 2 weeks and then randomly divided into different groups as desired. One week later, the mice were administrated with indicated treatments (*E. faecalis* EVs (50 ug in 200 ul PBS) or *E. faecalis* (1 x 10^8^ C.F.U resuspend in 200 μl PBS) or the control (200 μl PBS)) every other day until the end of the experiments. 1 × 10^6^ luciferase-expressing Hepa1-6-Luc cells were first injected subcutaneously into SPF C57BL/6 mice. After subcutaneous tumor formation, the tumor-bearing mice were euthanized, and the subcutaneous tumors were harvested. Well-vascularized and viable tumor edges were selected and cut into equal-sized pieces of approximately 1 mm^3^, which were then inoculated into the left lobe of mouse liver one week after the initiation of EVs gavage. Tumor burden was monitored using the IVIS Spectrum In Vivo Imaging System following intraperitoneal injection of D-luciferin (150 mg/kg/mouse). At the end of the experiments, the mice were sacrificed, and their livers were isolated and quantified using an IVIS Spectrum. Quantifications were performed with Living Image v.4.5.2.

**Immunohistochemistry staining**

After deparaffinization and rehydration, paraffin-embedded tissue sections (4 μm thick) were treated with retrieval buffer (pH 6.0 sodium citrate buffer) in microwave oven for 10 min. After cooling down, the sections were incubated in 3% H_2_O_2_ for 10 min to quench endogenous peroxidase activity and blocked with goat serum for 1 h. Then the sections were incubated with primary antibody overnight at 4 °C. The slides were then washed with PBS and incubated with secondary antibody at 37 °C for 30 min and stained with DAB substrate. The cell nuclei were stained with hematoxylin. Quantification of the percentages of indicated antibodies per area were performed using ImageJ. Five fields per tumor were chosen for quantification. All primary antibodies used in this study are listed in Supplemental Table 2.

**Cell culture and transfection**

HEK293T, Hep3B, SNU449, HUVEC, THLE-2 and Hepa1-6 cell lines were obtained from the American Type Culture Collection (ATCC), MHCC-97h cell line was lab storage. These cell lines were maintained at 37 °C and 5% CO_2_ (Thermo, Waltham, MA, USA). SNU449 were maintained in RPMI 1640 medium (RPMI). HEK293T, Hep3B, MHCC-97h, HUVECs and Hepa1-6 cells were maintained in Dulbecco’s modified Eagle’s medium media (DMEM). All the media were supplemented with 10%(v/v) fetal bovine serum (FBS) and 2 × 10^-3^ M L-glutamine. For transient transfection, plasmids were transfected into cell lines followed the standard protocol for Lipofectamine 2000 Transfection Reagent (Thermo Fisher, #11668019).

**Cell infection**

Cell infection by *E. faecalis* was performed as previously described^36^. Briefly, 2 days before infection, cells were seeded in triplicate in 6-well plates. Before infection, the cell culture medium was removed and the cells were washed once with PBS and incubated in serum-free medium for 2 h. *E. faecalis* was harvested and washed twice in PBS, and resuspended in medium without serum to be used at a multiplicity of infection (MOI) of 30. Infection was synchronized by 1 min centrifugation at 1000g. After 3 h of contact, cells were washed 5 times with PBS, and an antibiotic cocktail (150 μg/ml gentamicin and 10 μg/ml vancomycin) was added to kill extracellular bacteria. The efficiency of the antibiotic cocktails was controlled by the absence of viable colonies after plating of the cell supernatants.

**Cell viability assay and colony formation assay**

For cell viability assay, cells were seeded into 96-well plates at a density of 1000 cells per well. 10%(v/v) Cell Counting Kit-8 (K1080, Apexbio Technology LLC, Houston, TX, USA) solution was added to the well. After 3 h incubation, optical density at 450nm was measured using EpochTM Multi-Volume Spectrophotometer and Take3TM (BioTek, Winooski, VT, USA).

For colony formation assay, cells were seeded into 6-well plates at a density of 800 cells per well. After 7 to 10 days, cells were washed three times with PBS, the colonies were fixed with 4% polyoxymethylene and stained with 0.05% crystal violet at room temperature for 30 min. Number of colonies was calculated by Image J software.

**Tube formation assay and migration assay**

For the tube formation assay, matrigel matrix (Corning, USA) was first thawed at 4°C and spread into 96-well plates with 50 μl in each well and rested at 37 °C for 30 min to form a gel. 1×10^4^ pretreated (co-cultured with *E. faecali*s at MOI of 30 for 3 h, or 10% EF-CM for 24 h, or 50 μg/ml EVs for 24 h) HUVEC were suspended in 100 μl of the culture medium. After 16 h incubation, tube formation was photographed with a digital camera system, and the total length and number of branches of the tubes were analyzed with Image J software.

For the migration assay, pretreated 5 × 10^4^ HUVEC cell suspended in 200μm medium without FBS were seeded in the upper chamber (diameter = 8 μm; Corning Costar, New York, USA), and 600 μl medium containing 10% FBS were added to the lower chamber. After 24 h of incubation, cells were stained with 0.1% crystal violet for 30 min and 5 random fields of view were selected to count the number of migrating cells. Photographs were taken for counting with a digital camera system.

**RNA Seq Analysis**

RNA-Seq analysis was performed in Hep3B cells with or without infection with *E. faecalis* and 10% EFCM treatment. Total RNA extracted from the indicated groups of Hep3B cells was subjected to RNA-Seq performed by SHANGHAI BIOTECHNOLOGY CORPORATION (Shanghai, China). The sequencing reads were analyzed to obtain expression profiles. Gene set enrichment analysis (GSEA) was performed used the GSEA software provided by the Broad Institute (<http://www.broadinstitute.org/gsea/index.jsp>), in accordance with the instructions provided by the Broad Institute.

**Immunoblotting**

The immunoblotting was conducted as previously described^45, 46^. Total cell or tissue lysates were solubilized in lysis buffer (50 mM Tris–HCl, 1 mM EDTA, 150 mM NaCl, 0.1% NP-40, 0.1% Triton-100) containing protease inhibitors cocktail and phosphatase inhibitors (Bimake). Lysates were immunoprecipitated with indicated antibodies. Proteins were resolved by SDS-PAGE gels and then proteins were transferred to polyvinylidene difluoride membranes (Millipore). The membranes were blocked with 5% nonfat milk for 1 h at room temperature followed by incubation with indicated primary antibodies. Subsequently, membranes were washed in Tris-buffered saline with Tween-20 (Sangon Biotech, #A600669-0250) there times and incubated with indicated peroxidase-conjugated secondary antibodies (Thermo Scientific, #31430) for 1 hour at room temperature. Following several washes, chemiluminescent images of immunodetected bands on the membranes were recorded on X-ray films using the enhanced chemiluminescence (ECL) system (Bio Rad, #170-5061). The primary antibodies are listed in Supplemental Table 2.

**Anti-puromycin immunoblot analysis.**

Cells were plated in pre-warmed culture medium in sterile tissue culture plates and treated with the indicated treatment. After completing the treatments, the cells were washed twice with pre-warmed sterile PBS, and were cultured with 1 μM puromycin under normal culture conditions for 30 min. At the end of the incubation period, medium containing puromycin was aspirated, and the cells were washed three times with ice-cold PBS. The cells were then harvested to extract proteins for immunoblot assays. The PVDF membrane was incubated with anti-puromycin antibody.

**RNA isolation and quantitative real-time PCR.**

Total RNA was extracted from tissues and cells using TRIzol reagent (Invitrogen) and 1μg RNA was reverse transcribed to complementary DNA (cDNA) using ReverTra Ace® qPCR RT Master Mix with gDNA Remover (TOYOBO, Osaka, Japan) according to the manufacturer’s instructions. Quantitative real-time PCR analyses were performed using 2 × SYBR Green qPCR Master Mix (Bimake) and specific primers on a LightCycler 480 (Roche, Basel, Switzerland). All sequences for qRT-PCR used in this study are listed in Supplemental Table 5.

**Plasmids Construction and overexpression cell lines construction**

*Obg* cDNA was amplified by PCR from *E. faecalis* cells and cloned into PLVX vector and PCDNA3.1 with HA and MYC tag. The primers were listed in the Supplemental Table 3. For lentivirus preparation, HEK293T cells were seeded in a 10cm dish at a density around 1 × 10^6^, and were co-transfected with 10 μg PLVX-Obg plasmid, 5 μg psPAX2 and 5μg pMD2.G by using polyethyleneimine (Polysciences, 24765). The supernatant which contained lentivirus was collected at 48 and 72 h after transfection, and were filtered through Millex-GP Filter Unit (0.22 μm pore size, Millipore). Hepa1-6 cells were infected with filtered viral supernatant containing 10 μg mL^−1^ polybrene (Millipore, TR-1003-G), followed by puromycin selection and finally verified by western blot. PCDNA3.1-Flag-mTOR was kept in the lab.

**Coimmunoprecipitation (Co-IP)**

The coimmunoprecipitation was conducted as previously described^45, 46^. After indicated treatment, cells were lysed with cell lysis buffer (50 mM Tris-HCl pH 7.5, 1 mM EDTA, 150 mM NaCl, 0.1% NP-40, 0.1% Triton-100) containing protease inhibitors cocktail and phosphatase inhibitors (Bimake). For each lysate, supernatants were collected after centrifugation and incubated with appropriate antibodies overnight at 4°C, followed by Anti-Myc Magnetic Beads incubation for 4 h. After incubation, beads were washed with cell lysis buffer for three times. After that, add propriate volume of 2 × loading buffer to the beads and boiled for 10 min in 95 °C to get the eluted proteins. Next, immunoblot assays were performed with specific antibodies The primary antibodies are listed in Supplemental Table 2.

**In situ proximity ligation assay (PLA)**

The standard commercial protocol (Sigma-Aldrich, DUO92101) was followed for conducting PLA ^45^. The fixed HKT293T cells were subjected to permeabilizing in a 0.5% Triton X-100 solution for 10 min. Subsequently, PLA blocking solution was employed for blocking for 1 h prior to the incubation of the primary antibodies. After overnight incubation in a humidified chamber at 4 °C, the samples were treated with PLA secondary probe and incubated at 37 °C for 1 h. The ligation process was completed by applying Ligation mix to each sample, incubating them at 37 °C for 30 min. Polymerization mix was used for amplification and the samples were further incubated at 37 °C for 100 min. After incubation, 1 × Buffer B was used to wash the samples once for 10 min, followed by another wash with 0.01 × buffer B for 1 min at room temperature. Finally, the samples were prepared for imaging by mounting them with Duolink in situ mounting medium containing DAPI for 15 min. The proximity ligation signal was captured using a confocal microscope.

**Bacterial Attachment Assay.**

The bacterial attachment assay was performed as described previously. Briefly, 4 x 10^5^ cells were plated in a 6-well plate and co-cultured with *E. faecalis* (MOI = 10). After co-culture for 1 h, cell medium was removed and cells were washed with PBS three times. To lyse the cells, 200 µl of sterile H_2_O was added for 20 min, followed by the addition of 2 ml of BHI broth to homogenize the cells. The attached *E. faecalis* colonies were recovered on BHI agar plate. After 14 h, the number of colonies was counted.

**Doxycycline Inducible Cells Construction**

Lentiviral particles were generated by transfecting HEK293T cells with pLKO.1 shRNA (sequence listed in Supplemental Table S6.) or Tet-pLKO-puro vector construct and packaging vectors (psPAX2 and pMD2.G). Then the cancer cells were infected twice with culture medium containing lentivirus in the presence of 8 mg/mL polybrene. 48 h after infection, selection of transduced cells was performed with puromycin to increase the knockdown efficiency.

**The dual and multiplex immunohistochemistry.**

The dual and multiplex immunohistochemistry were performed using a four-color, three-plex immunohistochemistry kit (RC0086plus-34RM, Huilan Biotechnology, Shanghai, China). Briefly, the pre-treated cells were plated on the cell crawling slides. After fixation and permeabilization, the cell slides were subjected to quenching of endogenous peroxidase activity and serum blocking. The slides were then incubated with Obg antibody. After rinsing with PBS, the slides were incubated with an HRP-conjugated secondary antibody working solution. Subsequently, the slides were treated with the fluorescent dye, followed by an antibody eluent (RC-010, Huilan Biotechnology, Shanghai, China) treatment to eliminate nonspecific dye binding. The same protocol was applied for mTOR and LAMP1 staining. Cell nuclei were stained with DAPI. The slides were imaged using a confocal microscope system (Zeiss, Germany) and the resulting images were analyzed using Zen software.

**GTPases activity assay.**

EF-EVs and EF-dCas-Obg-EVs were extracted as previously described. The GTPase activity were assessed using a GTPase Activity Assay kit (Colorimetric) (P2435S, Beyotime Biotechnology, Shanghai, China). EVs were disrupted by ultrasonication at 40W for 30 cycles, and the protein concentrations were determined by BCA assay. Samples were diluted with Assay Buffer to different protein concentrations. GTP were added at different time points, mixed thoroughly, and incubated at 37°C. Chromogenic reagent was added, followed by incubation at 37°C, and absorbance at 630nm was measured.

**Obg protein purification.**

Briefly, the PET21a-Obg plasmids were transformed into *E.coil* BL21(DE3).1.5mL of the bacterial culture was induced with 1 mM IPTG overnight at 16°C with shaking at 200 rpm. The induced cells were harvested by centrifugation at 10,000g for 1min. The cell pellet was resuspended in lysis buffer (PBS pH 7.5, 10% Glycerol, 1 mM PMSF) and lysed by ultrasonication for 20 min. The lysate was centrifuged at 12,000g for 10 min, and the supernatant was collected while the precipitated was discarded. An appropriate amount of pre-equilibrated Ni-NTA resin was added to the supernatant and incubated at 4°C for 30 min with gentle shaking. The resin was then collected, and the flow-through was temporarily saved. The resin was washed and eluted with imidazole buffer of increasing concentrations using column chromatography.
